# Conserved piRNA Expression from a Distinct Set of piRNA Cluster Loci in Eutherian Mammals

**DOI:** 10.1371/journal.pgen.1005652

**Published:** 2015-11-20

**Authors:** Gung-wei Chirn, Reazur Rahman, Yuliya A. Sytnikova, Jessica A. Matts, Mei Zeng, Daniel Gerlach, Michael Yu, Bonnie Berger, Mayumi Naramura, Benjamin T. Kile, Nelson C. Lau

**Affiliations:** 1 Department of Biology and Rosenstiel Basic Medical Science Research Center, Brandeis University, Waltham, Massachusetts, United States of America; 2 Research Institute of Molecular Pathology, Vienna Biocenter, Vienna, Austria; 3 Mathematics Department and Computer Science and Artificial Intelligence Laboratory, Massachusetts Institute of Technology, Cambridge, Massachusetts, United States of America; 4 Eppley Institute, University of Nebraska Medical Center, Omaha, Nebraska, United States of America; 5 The Walter and Eliza Hall Institute of Medical Research, Parkville, Australia; University of Utah School of Medicine, UNITED STATES

## Abstract

The Piwi pathway is deeply conserved amongst animals because one of its essential functions is to repress transposons. However, many Piwi-interacting RNAs (piRNAs) do not base-pair to transposons and remain mysterious in their targeting function. The sheer number of piRNA cluster (piC) loci in animal genomes and infrequent piRNA sequence conservation also present challenges in determining which piC loci are most important for development. To address this question, we determined the piRNA expression patterns of piC loci across a wide phylogenetic spectrum of animals, and reveal that most genic and intergenic piC loci evolve rapidly in their capacity to generate piRNAs, regardless of known transposon silencing function. Surprisingly, we also uncovered a distinct set of piC loci with piRNA expression conserved deeply in Eutherian mammals. We name these loci Eutherian-Conserved piRNA cluster (ECpiC) loci. Supporting the hypothesis that conservation of piRNA expression across ~100 million years of Eutherian evolution implies function, we determined that one ECpiC locus generates abundant piRNAs antisense to the STOX1 transcript, a gene clinically associated with preeclampsia. Furthermore, we confirmed reduced piRNAs in existing mouse mutations at ECpiC-Asb1 and -Cbl, which also display spermatogenic defects. The Asb1 mutant testes with strongly reduced Asb1 piRNAs also exhibit up-regulated gene expression profiles. These data indicate ECpiC loci may be specially adapted to support Eutherian reproduction.

## Author Summary

Animal genomes from flies to humans contain many hundreds of non-coding elements called Piwi-interacting RNAs (piRNAs) cluster loci (piC loci). Some of these elements generate piRNAs that direct the silencing of transposable elements, which are pervasive genetic parasites. However, we lack an understanding of the targeting function for the remaining bulk of piRNAs because their loci are not complementarity to transposable elements. In addition, the field does not know if all piC loci are quickly evolving, or if some piC loci might be deeply conserved in piRNA expression, an indication of its potentially functional importance. Our study confirms the highly rapid evolution in piRNA expression capacity for the majority of piC loci in flies and mammals, with many clade- and species-specific piC loci expression patterns. In spite of this, we also discover a cohort of piC loci that are deeply conserved in piRNA expression from the human to the dog, a significantly broad phylogenetic spectrum of eutherian mammals. However, this conservation in piRNA expression ends at non-eutherian mammals like marsupials and monotremes. Existing mutations in two of these Eutherian-Conserved piC (ECpiC) loci impair mouse reproduction and abrogate piRNA production. Therefore, we suggest these ECpiC loci are conserved for piRNA expression due to their important function in eutherian reproduction and stand out as prime candidates for future genetic studies.

## Introduction

In animal RNA interference (RNAi) pathways, the Argonaute (AGO) and Piwi proteins are deeply conserved from humans to basal animals like cnidarians and poriferans [1, 2]. Likewise, several AGO-bound microRNAs (miRNAs) are also deeply conserved across bilaterians [3], but Piwi-interacting RNAs (piRNAs) differ by evolving so rapidly that there are few individual piRNAs conserved between even closely related species [4, 5]. A rationale for the fast rate of piRNA sequence evolution is to keep pace with the rapidly evolving transposable elements (TE) that some piRNAs are targeting [6, 7]. However, most mammalian piRNA cluster (piC) loci are depleted of TE sequences [4, 5], and in spite of the TE-repressing function of the Piwi pathway, animal genomes vary widely in size due mainly to TE content, from ~10% of the euchromatic genome in flies to >40% in mammals [8]. Thus, we have an incomplete understanding of what additional roles the Piwi pathway plays beyond TE repression.

The piRNAs arise as clusters from two main types of loci. Intergenic piRNA cluster loci are large 10–100 kb long regions, and some of these loci function to silence TEs with TE-directed piRNAs. However, the function of most intergenic piRNA cluster loci in mammals remains mysterious because they are depleted of TE sequences and the transcripts are non-coding [9]. In contrast, genic piRNA cluster loci are derived from protein-coding transcripts that possess extensive 3'Untranslated Regions (3'UTRs) which efficiently enter the piRNA biogenesis pathway [10, 11]. Hundreds of genic piRNA cluster loci are also depleted in TE sequences, and germ cells specifically select genic transcripts via an unknown signature for piRNA biogenesis [11]. Nevertheless, the abundant piRNAs from the *Drosophila* genic piC *traffic jam* (*tj*) may regulate gene targets important for follicle cell development [12].

Although piRNAs are essential for animal fertility, we do not know which of the hundreds of piC loci are most important for animal reproduction, particularly in mammals. Therefore, we hypothesize that determining which piC loci are most conserved in piRNA expression throughout animal evolution would yield functional insight to this question above. However, the field has only touched upon the evolutionary patterns of piC loci with a handful of previous studies comparing piC loci between a limited set of animal species. In Drosophilid flies, the intergenic piC locus *flamenco* (*flam*) is syntenically conserved between *D*. *melanogaster* (*D*.*mel*) and *D*. *erecta* (*D*.*ere*) for ~10 million years (MY) of evolution [13]. In rodents and humans, which diverged in evolution ~90 MY ago, some piC loci are also syntenically conserved [4, 5], and Assis and Kondrashov applied a classification process to these human and rodent piRNA datasets to conclude that piC loci were rapidly expanding at a high rate, with mainly new gains of clusters between these species and no piC loci losses [14]. Assis and Kondrashov further proposed a positive selection process for piC expansion to keep pace with the rapid evolution of TE sequences [14], however the capacity of piCs to expand via copy number evolution has recently been re-inspected in humans by Gould et al, whom suggest instead that negative selection may be acting upon human piCs to limit their copy number expansion [15].

Earlier studies only examined human, mouse and rat piRNA datasets that are now considered shallow by today’s deep-sequencing standards [4, 5], and thus were unable to determine whether piRNA expression from piC loci could be deeply conserved like miRNA expression. To gain a more complete picture of the evolution of piC loci expression, this requires the experimental support of new, deeper piRNA datasets from a broader spectrum of animals. Fortunately, the diversity of species with sequenced piRNA libraries have greatly expanded recently with these various studies [11, 16–26], some of which mainly focused on the characterizing the novel piRNA features specific to the species. Thus, we sought to discover: (1) which piC loci, if any, may exhibit deep conservation of piRNA expression across animal lineages, (2) how frequently do piC loci expression patterns vary between species, and (3) which gene regulation step(s) prominently influences these piC loci expression patterns. Here, we formally define conserved piC loci expression as the detection of mature piRNAs from syntenic loci between species, which is a process that depends on both: (1) the transcription of the loci, and (2) selection of the transcribed precursor RNA by Piwi-pathway proteins for processing into mature piRNAs.

In this study, we describe a comprehensive comparative genomics approach focusing first on the genic piRNA biogenesis mechanism that we had previously shown was deeply conserved between flies and mice [11]. Although gene homologs for mouse and fly genic piC loci can be predicted bioinformatically (S1A Fig), it is not clear if these are actually orthologous piC loci because gene families have greatly expanded in mouse during the >600 MY of evolution between these species. Thus, we decided to examine piRNA expression patterns within closely-related species amongst the Drosophilids and tetrapod lineages to determine how many piC loci expression patterns for piRNA biogenesis are deeply conserved. We confirm that genic piC loci, which are depleted in TE sequences, are rapidly evolving and expanding into repertoires that are unique to animal species. Surprisingly, we also discover a cohort of piC loci with piRNA expression patterns conserved across ~100 MY of Eutherian mammal evolution, which stand out from the generally rapid evolution of most animal piC loci expression patterns.

## Results

### Comparative genomics of genic piRNA cluster loci expression patterns

**Fig 1:**
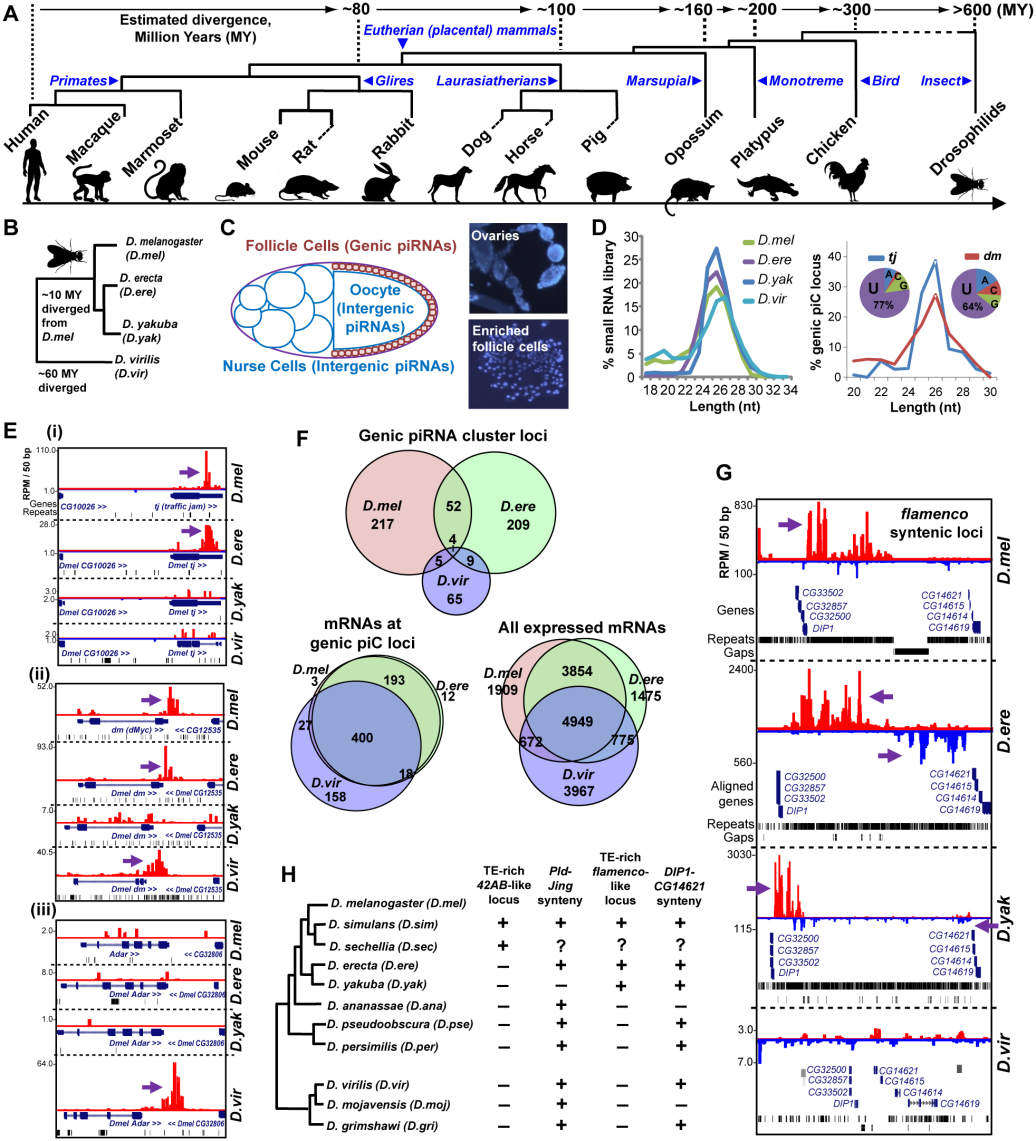
Comparative genomics of piRNA cluster (piC) loci and rapid evolution of piC loci expression patterns in Drosophilids. (**A**) Phylogenetic tree of the animals whose piRNAs are analyzed for this study. (**B**) Phylogenetic tree of the Drosophilids whose female piRNAs are profiled in this study. (**C**) Diagram of the Drosophilid ovarium where the different types of piRNAs are most abundantly expressed, and DAPI staining images of follicle cells enriched from ovaries during our preparations to balance the detection of genic piC loci from being overwhelmed by intergenic piC loci [28]. (**D**) Read length distributions for each small RNA library (left plot) and just the piRNAs in the genic piC loci, *tj* and *dm*. The pie chart insets show the 5' nucleotide composition of the genic piRNAs in these two loci.(**E**) Genome browser snapshots of the genic piC loci corresponding to piC-*tj* (**i**), piC-*dm* (**ii**, also known as *Drosophila c-Myc*), and piC-*Adar* (**iii**) showing some conserved and species-specific piRNA expression patterns. Purple arrows point to the start of bulk of piRNAs. Plus strand read peaks are red, minus strand read peaks are blue. (**F**) Venn diagrams of the overlap in genic piC locus expression (top) and the overlap in mRNA expression profiles (bottom sets) in three Drosophilid ovarium samples. The distributions of genic piC loci overlap are distinct from the distributions of mRNA expression profiles overlap (all *p<0*.*001*, Chi square test and ratio proportions test with Bonferroni correction). *D*.*yak* was omitted from this analysis because of much lower numbers of genic piC loci (see S2A Fig). (**G**) Snapshot of syntenic regions containing orthologous *flamenco* (*flam*) piC loci in *D*.*mel*, *D*.*ere*, and *D*.*yak*, but absence of this intergenic piC locus in *D*.*vir*. (**H**) Phylogeny of 11 Drosophilids with sequenced genomes and summary of the inspection in the genomes for the features at the syntenic regions for a *42AB*-like locus and a *flam*-like locus, as defined by piRNAs or a contiguous large block concentrated with TEs between the flanking genes. Plus indicates locus/feature presence, minus is absence, while the question mark indicates a major gap in the genome assembly preventing determination. See S3 Fig for additional genome browser snapshots.

We developed a piC loci discovery approach on new piRNA datasets that we had generated as well as analyzing publicly-deposited datasets covering four Drosophilids, the chicken and eleven mammals within Primate, Glire, and Laurasiatherian clades (Fig 1A and 1B). We sequenced Drosophilids piRNAs from ovarium samples because piRNAs are more plentiful and diverse in fly ovaries compared to fly testes [13, 27]. The tetrapod piRNAs were sampled from adult testes, which contained piRNAs from both early and post-pachytene stages of meiotic germ cells [19]. We generated a variety of piRNA libraries from Glires, including libraries enriched in pre-pachytene piRNAs from 10 days-post-partum (10dpp) testes and PIWIL2 immunoprecipitates (IPs), whereas the remaining tetrapod libraries were previously published in these studies: [11, 19–26]. This piRNA compendium was chosen for comprehensiveness and quality of piRNA coverage, since all libraries displayed the expected main read length distribution of 23–32 nucleotides and sufficient read depth (4–143 million reads, S1 Table). This phylogenetic spectrum of piRNA dataset allowed us to compare and determine the conservation of piC loci expression patterns spanning both short (<50 MY) and long (>150 MY) divergence times.

To search for conserved expression of piRNAs across potential piC loci between species, we focused first on genic piC loci because the protein-coding genes are clear proxies for assigning orthology between potential piC loci. We deployed a small RNA profiling and bioinformatics procedure that identified genic piC loci by tracking piRNAs from protein-coding transcript 3'UTRs (S1B Fig). This procedure was highly accurate in tracking piRNAs at specific genic piC loci, because the reads exhibited the correct length ranges of 24-28nt for Drosophilid piRNAs and 24-31nt for mammalian piRNAs, as well as a bias for uridine at the 5' nucleotide of the small RNAs (S1C–S1F Fig).

### Rapid evolution of piRNA expression amongst Drosophilid piRNA cluster loci

We examined conservation of piRNA expression from piC loci in four model Drosophilids where TE-directed piRNAs were previously characterized (Fig 1B) [13, 29]. *D*.*mel* protein-coding transcript alignments served as excellent proxies for transcripts in the incomplete draft assemblies of the *D*.*ere*, *D*.*yakuba* (*D*.*yak*) and *D*.*virilis* (*D*.*vir*) genomes. To prevent intergenic piC loci from overshadowing genic piC loci, we processed the ovarium samples to enrich for follicle cells and this improved genic piC locus detection (Fig 1C and 1D, [28]). We discovered >270 genic piC loci expressed in both *D*.*mel* and *D*.*ere*, 82 genic piC loci in *D*.*vir*, but surprisingly only 27 loci in *D*.*yak* (S2A Fig and S2 Table). The most abundant genic piC locus in *D*.*mel*, piC*-tj*, was only conserved in piRNA expression in *D*.*ere*, whereas the genic piC locus at *diminutive (dm)*, the *Drosophila* c-Myc oncogene homolog, was more deeply conserved to *D*.*vir* in piRNA expression (Fig 1E). Although we discovered plenty of piRNAs for the *D*.*yak* ortholog of the *flam* intergenic piC locus (Fig 1G), indicating the *D*.*yak* library sufficiently contained follicle-cell specific piRNAs, genic piC loci were still depleted in *D*.*yak*. We examined gene expression profiles in *D*.*yak* follicle cells for known Piwi pathway genes and new genes associated with PIWI in *D*.*mel* OSS follicle cells as well as Northern blot comparisons (S2C and S2D Fig), but these investigations were unable to explain the depletion of genic piC loci in *D*.*yak*.

Therefore, we only further considered the genic piC loci expression patterns in *D*.*mel*, *D*.*ere*, and *D*.*vir* (Fig 1F), and observed that most *Drosophila* genic piC loci were unique to a single species, such as the piC-*Adar* that is specific to *D*.*vir* (Fig 1E–1Eiii). In fact, the mRNA gene expression profiles in the ovarium samples shared much more similarity between these three Drosophilids than the genic piC loci piRNA expression patterns (Fig 1F). Furthermore, the top piRNA-producing genic piC loci from *D*.*mel* were also expressed as mRNAs in the other Drosophilid ovariums despite frequently losing the capacity to generate piRNAs (S2E Fig). Finally, we determined the gain and loss rates of genic piC loci in Drosophilids, with the highest rapid gain rates displayed in *D*.*mel* and *D*.*ere* (S2F Fig). These data suggest that Drosophilid genic piC loci evolved rapidly at the level of sequence elements within the genic transcripts rather than in the control of gene expression, thus leading to this diversity in genic piC loci expression patterns across species.

Given the compactness of Drosophilid genomes, a major proportion of intergenic piRNAs in *D*.*mel* derive from the *flam* and *42AB* piC loci, the two master control loci implicated in repressing TEs to maintain fertility in females [13, 30, 31]. Therefore, we turned our attention to examining these two major intergenic piC loci, and found both to be remarkably young evolutionary inventions (<12MY), having only recently arisen in the *melanogaster* subgroup (Fig 1G, S3 Fig). By tracking piRNA expression or an intervening TE-rich region, we detected signatures of piC-*42AB* orthologs in *D*.*simulans* (*D*.*sim*) and *D*.*sechellia* (*D*.*sec*), and *flam* orthologs in *D*.*ere* and *D*.*yak*. However, *piC-42AB* was absent from *D*.*ere* and *D*.*yak* genomes, and no *flam* orthologs were conserved beyond these species despite conserved gene synteny around *flam* and piC-*42AB* loci across ~40 MY of Drosophilid evolution (Fig 1H). Previous evolutionary studies suggested a selective advantage for Drosophilid piRNAs to silence TEs, but only up to a limit, when the host organism also tolerates the residing TEs [32–34]. Our data echoes this fluidity for the TE silencing function of major intergenic piC loci. Although they appear essential for TE repression and fertility, large intergenic piC loci as elements can arise and evolve as rapidly as the smaller genic piC loci.

### Most mammalian piRNA cluster loci have also evolved rapidly

**Fig 2:**
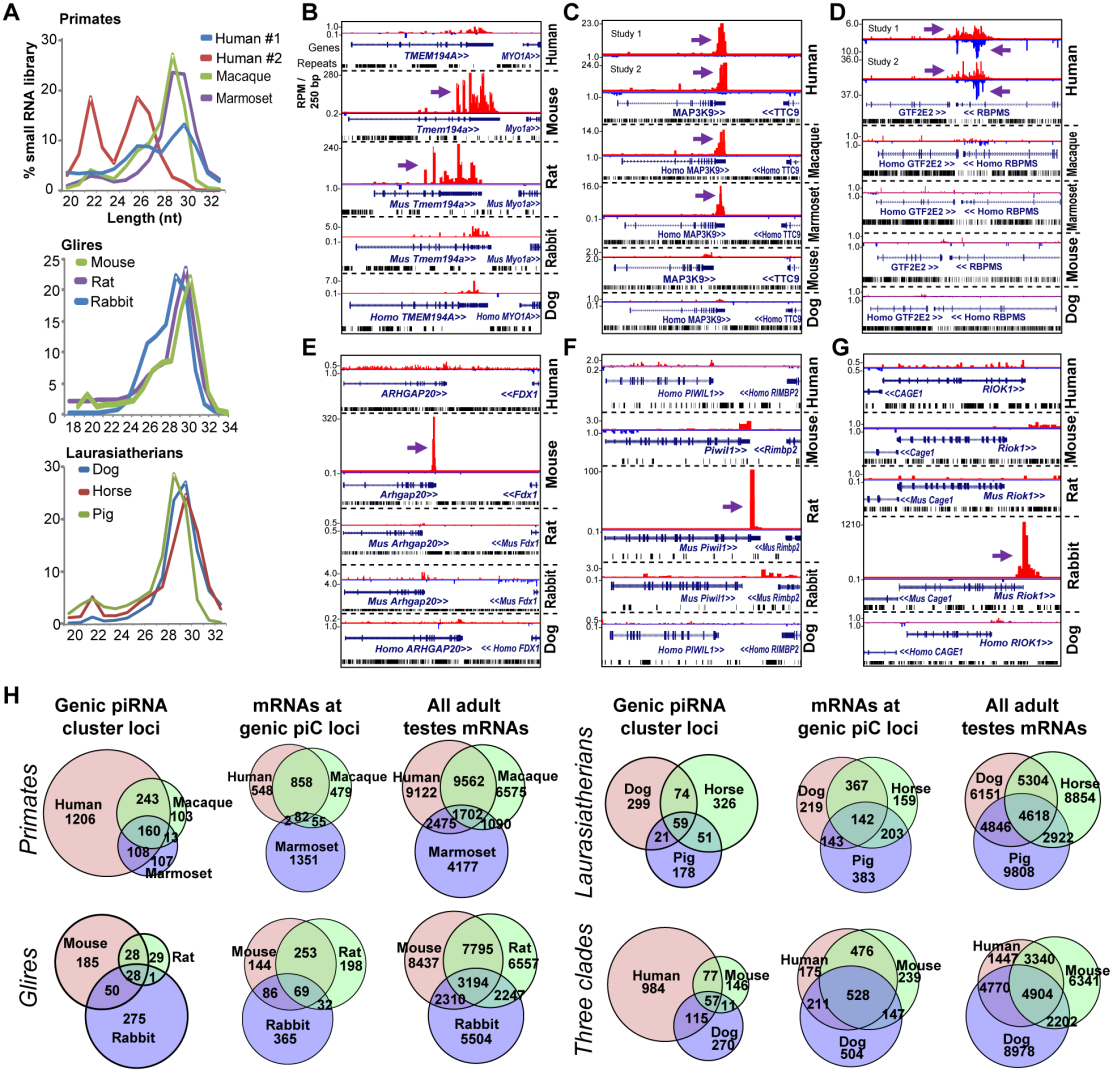
Rapid evolution of genic piC loci piRNA expression in mammals. (**A**) Read length distributions for each mammalian small RNA library analyzed for this study. All libraries are from adult testes total RNA, grouped according to Primates, Glires, and Laurasiatherians. Genome browser snapshots of the rodent-specific piC-*Tmem194a* (**B**); a primate-specific piC-*MAP3K9* (**C**); and two human-specific genic piC loci which have overlapping transcripts (**D**), piC-*GTF2E2* (plus strand) and piC-*RBPMS* (minus strand). The mouse-specific piC-*Arhgap20* (**E**); the rat-specific piC-*Piwil1* (**F**) and the rabbit-specific *piC-Riok1* (**G**). Purple arrows point to the start of bulk of piRNAs. Plus strand read peaks are red, minus strand read peaks are blue. (**H**) Venn diagrams of the overlap in genic piC loci expression (left) and the overlap in mRNA expression profiles (middle and right) amongst Primates, Glires, and Laurasiatherians, and a comparison species from the three clades. For each of species comparisons, the distributions of genic piC loci overlap are distinct from the distributions of mRNA expression profiles overlap (all *p<0*.*001*, Chi square test and proportions ratio test with Bonferroni correction).

Next, we applied our piC locus discovery approach to adult testes small RNA datasets from Primates (human, macaque, marmoset), Glires (mouse, rat, rabbit) and Laurasiatherians (dog, horse, pig), (Fig 2, S2 Table). We observed conserved piRNA expression for piC loci within Rodents (Fig 2B), and within Primates (Fig 2C). However, most genic piC loci, as defined by production of mature piRNAs, were uniquely detected in a single species, including human-specific genic piC loci that in two independent human testes studies appear to generate potentially overlapping 3'UTR-piRNAs (Fig 2D, S1F Fig). Although endogenous overlapping small RNAs are prevalent in invertebrates [35], this configuration of two potentially overlapping human piRNA cluster loci may hint to the existence of dsRNAs in the mammalian germline beyond endo-siRNA transcripts in mouse oocytes [36–38].

We examined three species-specific piC loci (*piC-Arhgap20*, *piC-Piwil1*, and *piC-Riok1)* that were only expressed in mouse, rat, and rabbit, respectively (Fig 2E–2G), and we confirmed these species-specific piRNA expression patterns by Northern blotting (S4A Fig). We also considered whether different stages of testes development or partitioning of piRNAs into the different mammalian Piwi proteins could be the root of these species-specific detections of genic piC loci (see S4B–S4H Fig and Supplementary Text Discussion). Despite comprehensive profiling of genic piC loci across PIWIL2 IP’s and different stages of Glire testes, the genic piC loci repertoires remained highly diverse between species. With regards to intergenic piC loci, their definitions amongst species were greatly influenced by the completeness of the species’ genome assembly, such that mouse and human exhibited the greatest number of intergenic piC loci (S3 Table). Nevertheless, at least 70% or more of genic piC loci could be reproducibly called by our pipeline in two independent human and chicken piRNA libraries (S4I Fig). These analyses mitigate the concern of limits in our piC discovery approach and confirm that the diversity in piC loci expression patterns between species is not an artifact.

Our approach was most effective in *D*.*mel*, human and mouse, where we detected the expression of >290, >1700, >2900 genic piC loci, respectively (Figs 1F, 2H and S4H). Considering the extensive piRNA sequencing coverage in human and mouse libraries, the comparison of piC loci expression patterns from all samples between these two species still show many species-specific genic piC loci expression patterns, which is also reflected by the Primate-specific and Glire-specific genic piC loci patterns (S4G Fig, middle, right). In addition, we observe within each of the three different clades (Primates, Glires, and Laurasiatherians) a tendency for adult testes gene expression profiles to show greater overlap when contrasted to the diversity of genic piC loci (Fig 2H). These patterns persisted even when we raised the stringency of cutoffs by 2-fold for piRNAs needed to call a shared piC locus expression and for gene expression profiles to be called overlapping (S2B and S4H Figs).

Amongst the top piRNA-generating genic piC loci in mouse and human, the gene orthologs that lost the capacity to make piRNAs in the most distant relative within the clade (rabbit and marmoset, respectively), still tended to be expressed in the adult testes as mRNAs (S4J and S4K Fig). Therefore, we propose that clade-specific and species-specific piC locus expression is a common feature in both flies and mammals; and that the rapid evolution of piC loci expression patterns is occurring at the transcript sequence level to alter entry into piRNA biogenesis pathways rather than at the transcript expression level. We also measured the rate of genic piC loci gain and loss throughout the phylogeny of tetrapods examined in this study, and consistently observed bursts of genic piC loci gains and very few losses since the number of ancestral genic piC loci were low (S4L Fig). This result is consistent with the gain and loss rates for genic piC loci in Drosophilids and further validates the earlier proposal that piC loci expansion is a common phenomenon [14].

### Sequence signatures and evolutionary rates of genic piRNA cluster loci

Despite rapid evolution of piC loci expression patterns between species, we wondered if analyzing genic piC loci sequences within fly and mouse genomes could reveal sequence motifs and structured RNA elements that might differentiate genic piC loci from standard mRNAs that do not generate piRNAs. To test this question, we selected the top piRNA-producing transcripts from the fly (*D*.*mel*) and the mouse, respectively, and also selected a list of negative control transcripts that had similar annotated 3'UTR lengths to genic piC loci yet did not make piRNAs (S5A Fig and methods). We then searched each list with an RNA feature analysis against the Open Reading Frames (ORF) and 3'UTR sequences. First, we counted the average number of significant structured RNA elements predicted genome-wide by the RNAZ, EvoFold and REAPR algorithms [39–41]. Compared to the negative control sets, the top set (highest piRNA-producing set) of fly genic piC transcripts appeared to be enriched in predicted structured RNA elements in the ORF sequences compared to the negative control set, whereas the 3’UTRs of top set of mouse genic piC transcripts were more enriched in structured RNA elements (S5B and S5E Fig). Two different sequence motif discovery programs, MEME and GLAM2 [42], both predicted a Poly-U-rich motif with greater statistically-significant enrichment amongst the 3'UTRs of both fly and mouse genic piC transcripts compared to the negative control sets, whereas no clear motif was enriched amongst the ORF’s. These initial surveys hint at intrinsic features that may distinguish a genic piC transcript from a regular protein-coding mRNA, and these features may frequently arise or disappear during animal evolution to yield the diversity of clade- and species-specific sets of genic piC loci.

To examine whether genic piC loci were subjected to different evolutionary forces compared to non-piRNA producing control transcripts, we compared sequence conservation and evolution rates for fly and mouse genic piC loci with normalized values of the phastCons [43] and phyloP [44] scores for ORF and 3'UTR sequences. High average phastCons scores reflect stronger conservation [43], whereas positive phyloP scores suggest selective constraints on the sequences’ evolution [44]. Our analyses suggest that genic piC ORFs with the greatest number of piRNAs in both flies and mice were more conserved and under greater selective constraint than negative control mRNAs (S5F and S5G Fig). The faster evolving 3'UTR sequences were expected to be less conserved, reflecting 2–5 fold lower normalized phastCons scores in 3'UTRs compared to ORFs, and these 3’UTRs displayed phyloP scores that were positive but not particularly high. In addition, the 3'UTR phastCons and phyloP scores were not statistically distinct between genic piC loci and negative control transcripts. Since the bulk of genic piRNAs derive from the 3'UTR, this result is consistent with the overall poor conservation of individual piRNA sequences between animal species.

### A set of piRNA cluster loci have conserved piRNA expression in Eutherian mammals

**Fig 3:**
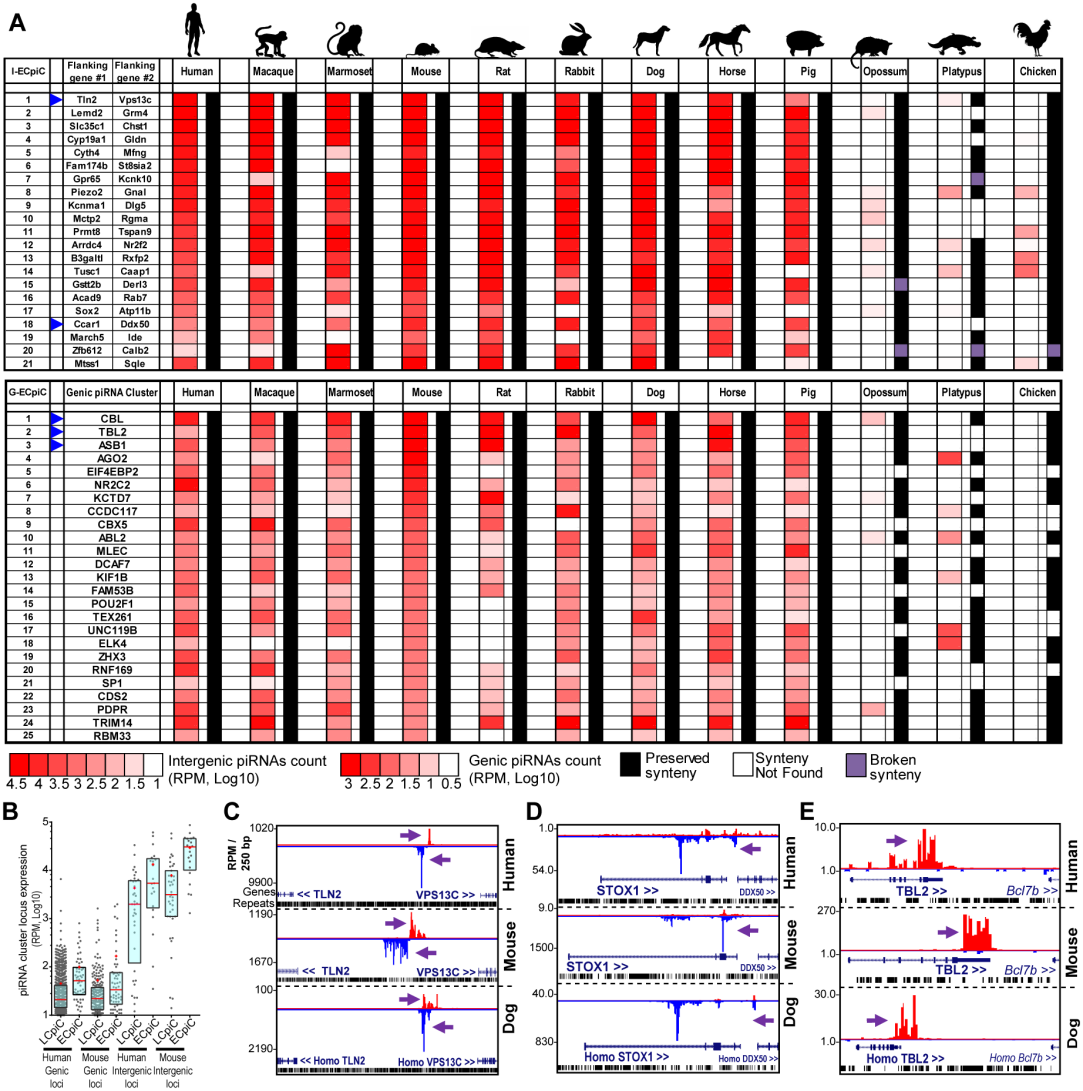
Eutherian-Conserved piRNA cluster (ECpiC) loci. (**A**) Heatmap diagram of piRNA expression and gene synteny for intergenic ECpiC loci (top) and the top 25 of 56 genic ECpiC loci (bottom). Remaining genic ECpiC loci and genomic coordinates for intergenic ECpiC loci are shown in S3 Table. Blue triangles highlight the piC loci discussed further in the study. (**B**) Box plots of interquartile ranges of piC loci piRNA expression levels. The red line and cross mark the median and mean, respectively. ECpiC loci tend express more piRNAs than Less-Conserved piC (LCpiC) loci. Genome browser snapshots of two notable intergenic ECpiC loci (**C, D**), and one notable genic ECpiC locus (**E**) from a representative of Primates (human), Glires (mouse) and Laurasiatherian (dog). Purple arrows point to the start of bulk of piRNAs. Plus strand read peaks are red, minus strand read peaks are blue. Full compilations are in S6 Fig.

Although most piC loci are rapidly evolving, we were struck by the conserved expression of 8% and 5% of genic piC loci in human and mouse, respectively (Fig 2H). To look for deeper conservation of piC loci expression beyond the ~80 MY of divergence between humans and mice, we discovered and compared piC loci expression patterns across nine mammals and the chicken, thus spanning ~300MY of tetrapod evolution [45]. This search revealed 21 intergenic and 56 genic piC loci conserved in piRNA expression across Primates, Glires, and Laurasiatherians (Fig 3), for which these three clades had diverged from a common Eutherian (placental) mammalian ancestor ~100 MY ago [46]. We name these piC loci as Eutherian-Conserved piRNA cluster (ECpiC) loci, which tended to yield many more total piRNAs per loci compared to the other Less-Conserved piRNA cluster (LCpiC) loci (Fig 3B).

**Fig 4:**
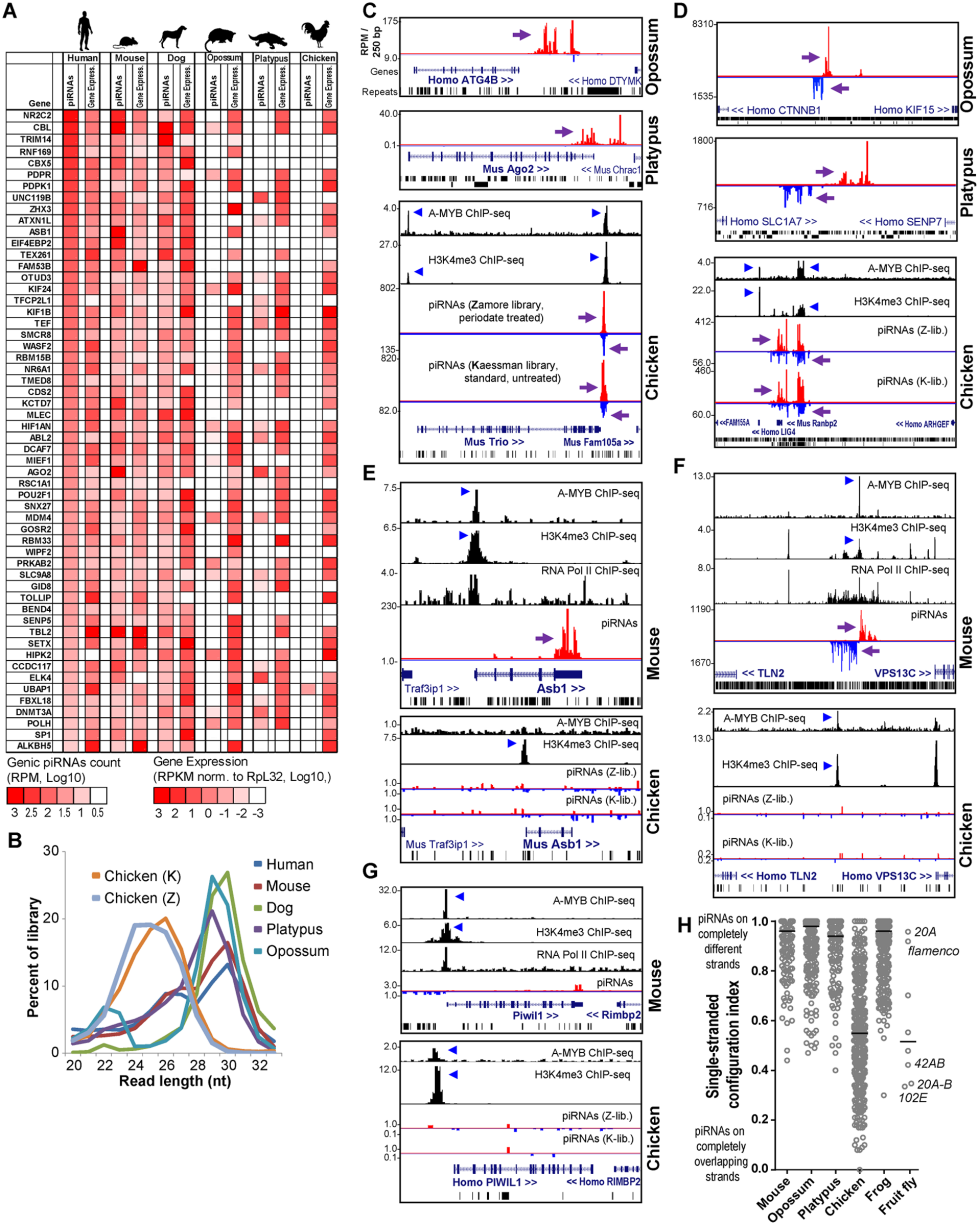
Comparison of opossum, platypus and chicken piC loci to mouse piC loci. (**A**) Heatmap showing piRNA and gene expression levels for genic ECpiC loci compared to the orthologs in opossum, platypus and chicken, for which many lack piRNAs but the mRNA transcript is consistently expressed in the adult testes. (**B**) Read length distributions for each tetrapod small RNA library analyzed for this study. All libraries are from adult testes total RNA. (**C**) Genome browser snapshots of genic piC loci from opossum (top), platypus (middle), and chicken (bottom). Purple arrows point to the start of bulk of piRNAs. Plus strand read peaks are red, minus strand read peaks are blue. (**D**) Intergenic piC loci from opossum (top), platypus (middle), and chicken (bottom). Comparison of piRNA and chromatin mark differences between the mouse *piC-Asb1* locus and the orthologous chicken Asb1 locus (**E**); the mouse intergenic ECpiC#1 and the syntenic region in chicken (**F**); and the mouse PIWIL1 locus to the chicken PIWIL1 locus (**G**). The blue triangles point to notable peaks in the ChIP-seq chromatin marks plotted from the data of Li et al [19]. (**H**) A plot of unique-strand configurations for the transcripts within major piC loci between representative mammals, chicken, frog and fly. Notable *D*.*mel* single-strand piC loci (index closer to 1) and dual-strand piC loci (index closer to 0) from *D*.*mel* are marked. The bar marks the median of the distribution.

Surprisingly, ECpiC loci were not consistently expressed in the opossum, a marsupial; and the platypus, a monotreme; which had diverged even further from Eutherian mammals at an additional ~60 and ~100 MY ago, respectively [45]. This is striking given that the synteny of genes around the ECpiC loci was still preserved, persisting from Eutherian mammals out to the chicken (Fig 3A). The mRNAs for the opossum, platypus, and chicken genes orthologous to genic ECpiC loci were also robustly expressed in the adult testes despite lacking the ability to generate piRNAs (Fig 4A). This suggests that the absence of conserved piRNA expression from the ECpiC loci in opossum, platypus, and chicken testes are not due to gene expression profile differences compared to Eutherian mammal testes. Furthermore, piRNA coverage in the opossum, platypus and chicken adult testes small RNA libraries was excellent and unobscured by miRNAs (Fig 4B)[19, 20]. Finally, we readily identified genic and intergenic piC loci in opossum, platypus, and chicken despite their draft genome states in the UCSC Genome Browser [47] (Fig 4C and 4D). Together, these results confirm ECpiC loci are only conserved in piRNA expression in Eutherian mammals, and suggest that the evolutionary conservation of ECpiC loci is due to preserving sequence elements directing piRNA biogenesis rather than piRNA cluster precursor transcript expression.

To further evaluate whether transcriptional regulation determines the choice of transcripts to become piRNA precursors, we considered a previous study showing the A-MYB transcription factor is conserved from mouse to chicken in binding to promoters of Piwi pathway genes to promote a feed-forward loop of piRNA biogenesis in the mouse and chicken testes (i.e. A-MYB peak at Piwil1 [19], Fig 4G). Although we observed many clear A-MYB peaks at the putative promoters of both chicken and mouse piC loci (Fig 4C–4F), there were notable examples of piRNA absence despite transcriptional activation, such as conserved A-MYB binding and H3K4me3 peaks in a chicken locus orthologous to mouse intergenic ECpiC#1, and conserved A-MYB and H3K4me3 peaks at mouse Piwil1. However, few piRNAs were detected from the intergenic piC syntenic chicken locus or from mouse and chicken Piwil1 (Fig 4E–4G). This contrasts with ample Piwil1 genic piRNAs in the rat (Fig 2F). We conclude that transcriptional activation in tetrapod testes, including by A-MYB, is insufficient to determine piRNA biogenesis. This evidence further supports our hypothesis that the evolution of piRNA biogenesis signatures is occurring within the sequence of the precursor RNA transcript rather than in the control of transcription initiation.

Although opossum and platypus piRNA clusters were configured similarly to mammalian piRNA clusters for piRNA biogenesis from non-overlapping transcripts (Fig 4D and 4F), chicken piC loci were surprisingly distinct in their configuration of piRNA biogenesis compared to mammalian piC loci. Mammalian piRNAs typically map only to single strands such as single-stranded mRNA transcripts in genic piC loci or to two transcripts that emanate as bi-directional non-overlapping strands from a common promoter region for some intergenic piC loci [4, 5, 19, 48] (Fig 4F). Many chicken piRNAs instead mapped to both plus and minus strands for multiple genic and intergenic piC loci (Fig 4C and 4D). When we calculated a single-stranded configuration index for the major piC loci from three mammals, the chicken, the frog and the fly *D*.*mel* (Fig 4H), this index accurately distinguished the single-stranded *flam* and *20A* piC loci from the *42AB* and *102E* piC loci in the fly, and clearly confirmed that piC loci in all three mammals are predominantly single-stranded in their configurations. Interestingly, chicken piC loci displayed a wide range of piC configurations, with many more double-stranded piC loci with indexes similar to the fly *42AB* and *102E* double-stranded piC loci. Although the frog is evolutionarily a more distant tetrapod than chicken (~370 vs ~320 MY, respectively) in relation to humans [45], the frog piC loci determined from oocytes were surprisingly more single-stranded and similar in configuration to mammalian piC loci [49] (Fig 4H). These analyses and the shorter piRNA length distributions of chicken piRNAs (Fig 4B) strongly suggest that chicken piRNA biogenesis pathways may be more similar to flies compared to other tetrapods.

### Developmental functions for Eutherian-Conserved piRNA cluster loci

We hypothesized that extensive conservation of piRNA expression patterns across ~100 MY of evolution implies conserved developmental functions for ECpiC loci. A likely conserved function for intergenic ECpiC loci would be to generate particularly essential TE-directed piRNAs, but TE sequences are neither more enriched nor more conserved in ECpiC loci versus LCpiC loci (S3 Table), and mouse genetic studies knocking out parts of intergenic piC loci are only now beginning to emerge [50]. However, a compelling non-TE repression role may be associated with intergenic ECpiC #18 that is located between the CCAR1 and DDX50 genes and generates many piRNAs antisense to the STOX1 transcript (Fig 3C, S6 Fig). ECpiC#18 is an intergenic piC locus because its transcript is antisense to STOX1, has lower coding potential than genic piC loci, and H3K4me3 ChIP-seq patterns suggests transcription begins in the intergenic region between STOX1 and DDX50. The STOX1 gene is implicated as a genetic factor linked to the placental-based disease of preeclampsia in humans [51–53].

**Fig 5:**
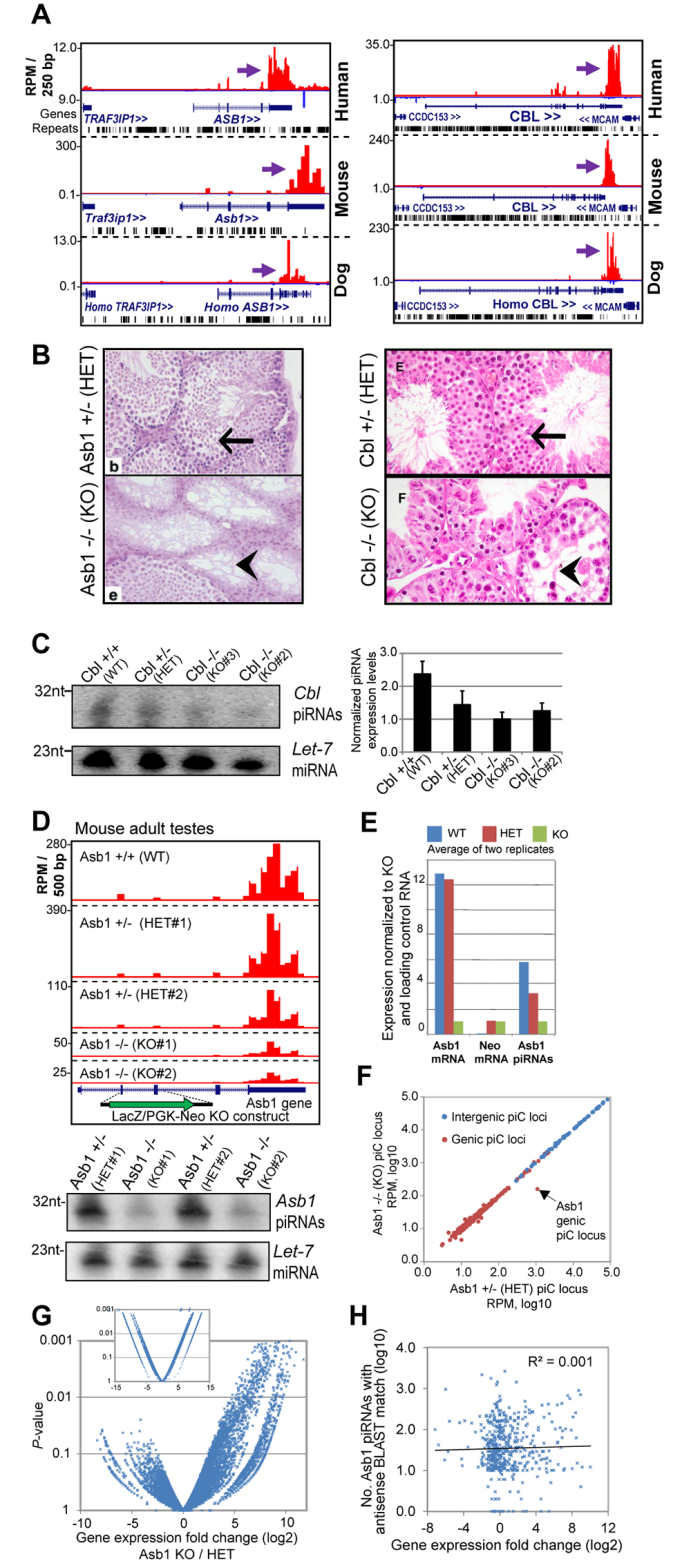
Phenotypes of two existing mouse mutants for genic ECpiC loci. (**A**) Genome browser snapshot of *piC-Asb1* (left) and *piC-Cbl* (right) in human, mouse and dog. Purple arrows point to the start of bulk of piRNAs. Plus strand read peaks are red, minus strand read peaks are blue. Full compilations are in S6 Fig. (**B**) Histology of mouse adult testes seminiferous tubules from the Asb1 mutant (left, image adapted from Fig 3 of [55], copyright of the American Society for Microbiology); and the Cbl mutant (right, image adapted from Fig 8 of [57], copyright of the Rockefeller University Press). Arrows points to normal spermatogenesis, the arrowheads points to empty tubules with sperm loss. HET, heterozygote, KO, homozygous knockout. (**C**) Northern blot analysis of Cbl piRNAs from adult testes from the mouse strain where Cbl exon 2 is flanked by LoxP sites (WT), or deleted by Cre recombinase for 1 allele (HET) or both alleles (KO). Quantitation of technical triplicates is shown to the right. (**D**) Top, profiles of Asb1 piRNAs from WT, HET and KO mutants which contain a LacZ/PGK-Neo insert disrupting exons 2 and 3. Two sets of animal pairs were examined. Bottom, Northern blot of Asb1 piRNAs and the Let-7 miRNA as loading control. (**E**) Quantitation of Asb1 piRNAs and Asb1 and Neomycin mRNAs. (**F**) Comparison of all piC loci expression between Asb1 KO and HET testes. (**G**) Volcano plot of gene expression profiles from testes (large plot) and kidney (smaller inset plot, same axis proportions). (**H**) Scatterplot of genes with predicted antisense-matching Asb1 piRNAs compared to gene expression changes between Asb1 KO and HET testes. The list of these gene names are in S4 Table.

A previous Gene Ontology analysis of mouse genic piC loci displayed an enrichment of gene function processes such as nucleic acid metabolism, transcription and regulation-related processes [11]. Such functions are also known for the genes that are ECpiC loci, such as the oncogenic transcription factors ABL2 and ELK4, the miRNA effector AGO2, and TBL2 and KCTD7 genes that are in the deleted locus of Williams-Beuren syndrome patients [54], which display developmental defects. While future genetic studies are required to investigate broader sets of ECpiCs, we looked for existing mutations in ECpiC loci that displayed developmental phenotypes and wondered if the mutations disrupted piRNA biogenesis. We noticed two prominent genic ECpiC loci corresponding to genes Cbl and Asb1 (Fig 5A). Knockout (KO) mice in each of these genes were created more than a decade ago because the genes were highly expressed in the blood [55, 56]. However, these mutants exhibited no major abnormalities, with the exception of being hypofertile. This defect was in both cases due to depleted spermatogenesis [55, 57] (Fig 5B).

Could the mutations in Cbl and Asb1 be causing a loss of genic piRNAs in the testes that might explain the spermatogenic defects? Northern blotting revealed that Cbl piRNAs were reduced ~2-fold in Cbl Heterozygotes (HET) and KO mutants when compared to the Wild-type (WT)(Fig 5C), which coincides with difficulties breeding both HET and KO Cbl mutants [57]. Next, we resurrected from sperm the original Asb1 mutant mouse line and sequenced adult testes small RNA-seq and mRNA-seq libraries. Indeed, we found that the LacZ-PGK-Neo insert disrupting the Asb1 transcript greatly reduced Asb1 piRNAs ~6-fold in the KO and ~2-fold in the HET testes (Fig 5D and 5E). Other genic and intergenic piC loci were unaffected in the Asb1 KO testes compared to HET testes (Fig 5F), indicating the specificity of the mutation only affecting the piC-Asb1. Expression profiling indicated a significantly greater number of up-regulated genes than down-regulated genes when comparing Asb1 KO testes to HET testes (Fig 5G), whereas far fewer genes were either up- or down- regulated in KO versus HET kidney, a somatic tissue expressing Asb1. In addition, no mammalian TE transcripts were detected to be up-regulated in the Asb1 mutant testes. Some but not all predicted targets complementary to Asb1 piRNAs were up-regulated in Asb1 KO versus HET testes (Fig 5H), so future studies will be focused at distinguishing which up-regulated transcripts in the Asb1 mutant are direct or indirect targets of the piRNAs.

## Discussion

Our study provides a new understanding of the evolutionary patterns for animal piRNAs and piC loci. These integral components of the ancient Piwi pathway evolve much more quickly than RNAi protein factors and other small regulatory RNAs like miRNAs, which can be conserved as far back as ~500 MY of evolution [1, 2]. By matching orthologous piC loci via synteny and protein orthology, and then broadly profiling piRNAs and mRNAs across animal gonads, we confirm rapid evolution in the piRNA expression patterns for both genic and intergenic piC loci even between close relatives within insect and mammalian clades that have only diverged by ~10 MY of evolution. This rapid gain of species-specific genic piC loci in both insects and tetrapods is consistent with a previous study’s proposal that piC loci are expanding rapidly via positive selection processes [14]. However, Eutherian mammals have distinctly conserved the expression of piRNAs from a notable set of ECpiC loci through ~100 MY of evolution, whereas very few deeply conserved piRNA expression patterns were observed in flies.

What might explain the lack of deeply-conserved piRNA expression patterns in flies? Perhaps piC loci evolution may be accelerated in Drosophilids due to certain aspects of the Piwi pathway that are currently unique to *Drosophila*, such as the capacity of *de novo* TE insertions to promote new piRNAs from flanking genomic sequence [58, 59], *Drosophila*-specific piRNA biogenesis factors like RHINO, CUTOFF, and DEADLOCK [58, 60–62], and epigenetic induction and suppression of piRNA biogenesis [63–68]. In addition, whereas only a minority of mammalian piRNAs are complementary to TEs [4, 5], the majority of *Drosophila* piRNAs are complementary to TEs [13, 30, 31], perhaps reflecting a more dynamic “arms race” between TEs and Drosophilid piRNAs [32–34].

Nonetheless, the rapid evolution for the majority of piC loci in both mammals and flies may also suggest that many piC loci are evolving by non-adaptive evolutionary forces that result in diverse piRNA repertoires, which germ cells may simply tolerate along with their highly diverse transcriptomes [69]. In addition, this diversity of piC loci between species may be attributed to higher frequencies of mutations in non-coding portions of genes and intergenic regions that allow transcripts to enter or leave the piRNA biogenesis pathway with greater frequency, since these turnover events may be under relaxed evolutionary constraints [14]. Nevertheless, some specific piC loci might also behave differently from the bulk of piC loci with regards to evolutionary constraints, as has been proposed for some human piC loci [15, 70]. Thus, the conservation of piRNA expression from ECpiC loci is striking, and we speculate these loci may have been selected to yield high levels of specific piRNAs in order to promote gene expression profiles favoring spermatogenic and embryonic fitness.

**Fig 6:**
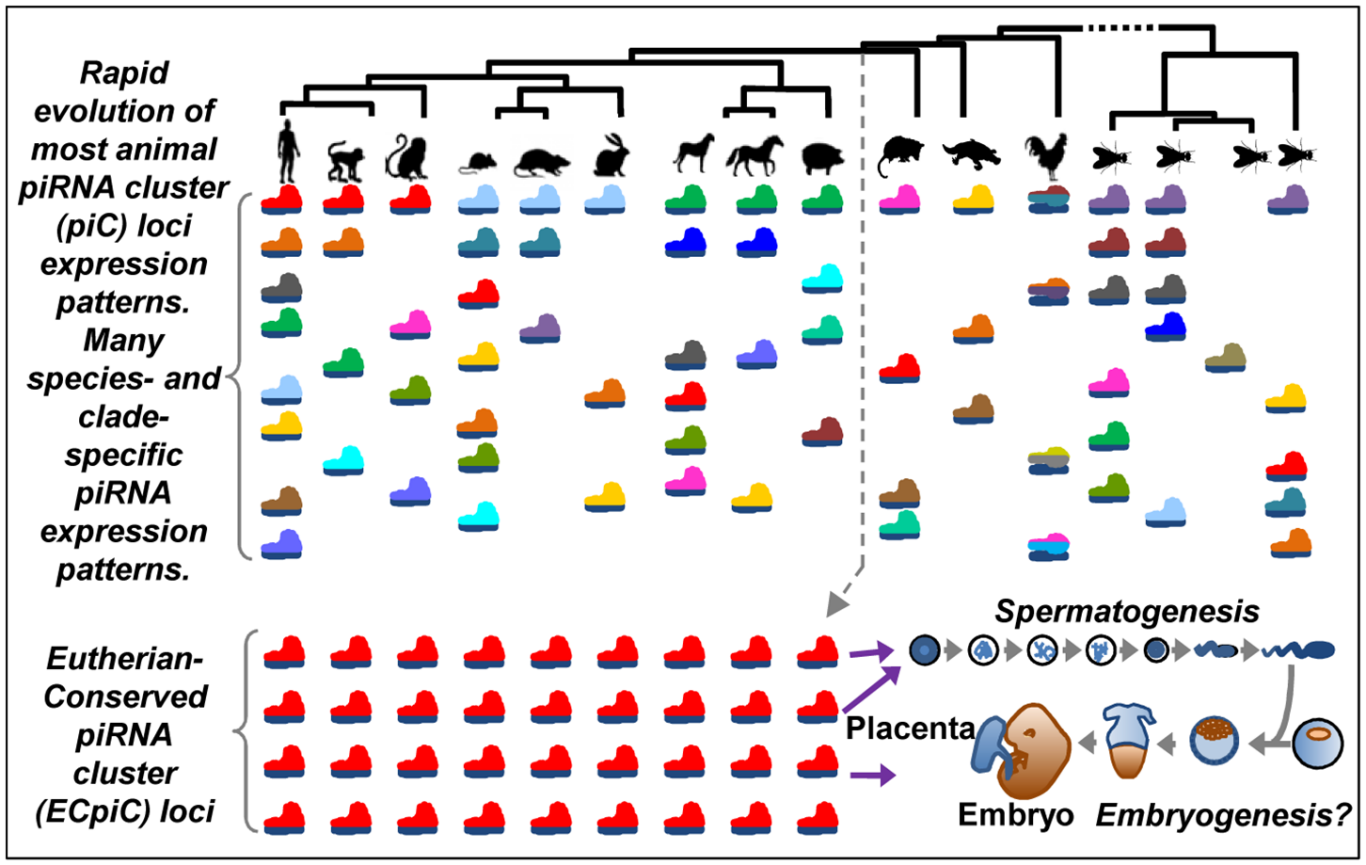
A model for the evolution of piC loci expression patterns. The rainbow of colored pictograms represents the diverse number of piC loci that are generally distinctly expressed between animal species or clades. We propose that most piC loci have evolved rapidly under non-adaptive evolutionary forces to result in this great diversity of piC loci expression patterns. However, a distinct set of piC loci (the same red-colored pictograms) is conserved in piRNA expression for >100M years of evolution in Eutherian mammals, perhaps to serve roles in mammalian reproduction.

This list of ECpiC loci will help us prioritize which loci to generate future piC loci mutants, and leads us to wonder if their functions will be tied to unique aspects of Eutherian reproduction, such as placental development and paternal genome regulation during spermatogenesis. Our analysis also suggests that most piC loci in vertebrates and flies are evolving under possibly non-adaptive evolutionary forces, whereby genetic drift may frequently create species- and clade-specific sequence motifs or RNA structural elements that allow diverse repertoires of transcripts to frequently enter (and exit) the piRNA biogenesis pathway. Although animal gonads are notable for their generally promiscuous transcriptional activity as well as rapid evolutionary turnover of genes [71, 72], we still observed that gene expression profiles between species are more similar than genic piC loci piRNA expression profiles (Figs 1 and 2). We propose a model for most piC loci being neutral for gonadogenesis fitness, and the plethora of individual piC loci may result in functional redundancy and allow fluidity in the emergence and evolutionary turnover of piC loci (Fig 6). However, some piC loci have been subjected to adaptive evolutionary forces in order to help germ cells suppress TE mobilization, such as prominent intergenic piRNA cluster loci serving as master TE control loci in Drosophilids [30].

Mutations disrupting Asb1 and Cbl in the mouse appear to also disrupt the piRNAs from these individual piC loci, hinting that the loss of piRNAs may be causing the spermatogenic defect. Indeed, more genes are up-regulated than down-regulated in the Asb1 mutant testes, with several up-regulated genes having complementarity to Asb1 piRNAs (Fig 5). Although we cannot exclude the idea that loss of Asb1 protein function is causing the spermatogenic defect, we note that five other close Asb1 homologs (Asb3, -4, -5, -8, and -9) are still highly expressed in the Asb1 mutant and might provide protein function compensation [55], whereas only Asb1 generates an abundant cluster of piRNAs. Future efforts should be directed at creating new mouse mutants that replace the endogenous 3'UTRs of Asb1 and Cbl with an equivalently long 3'UTR of a different gene that is also expressed highly in the germline but does not generate piRNAs. This approach may be better than simply deleting the endogenous 3'UTRs because Asb1 and Cbl mRNA stability and translation would likely be strongly diminished if they completely lacked a 3'UTR.

Our discovery of ECpiC loci suggests new regulatory functions beyond TE repression to accommodate specific aspects of Eutherian reproduction. There is a precedent for an epigenetic process specific to Eutherian reproduction, such as random X-chromosome inactivation via the *Xist* non-coding RNA, which differs from the paternally imprinted inactive X chromosome in marsupials and stochastic dosage compensation in monotremes [73]. ECpiC loci such as piC-Asb1, piC-Cbl, and ECpiC#18 may represent new gene targets to examine for cases of hypo-fertility, which may be more likely to persist in a population than completely sterile mutations. Albeit reduced in fecundity, reproduction may be still viable in young piC locus mutant animals, but germ cell longevity may also be limited. When new ECpiC loci mutants are made that will only disrupt the piRNAs while leaving the protein coding gene intact, we will be better able to discern which target genes are mis-regulated during spermatogenic decline or defects on placental development. Since the piRNAs from ECpiC#18 are mainly antisense to the STOX1 mRNA, a gene clinically implicated in placental development [51–53], we speculate that mis-regulation of ECpiC#18 might likely impact STOX1 expression and contribute to sperm phenotypes that fall within a hypothesis for paternal factors in the etiology of preeclampsia [74]. Future characterization and genetic studies in mouse will be useful to determine if ECpiC#18 directly regulates the STOX1 locus.

## Materials and Methods

### Ethics statement

This study was performed in strict accordance with the recommendations in the Guide for the Care and Use of Laboratory Animals of the National Institutes of Health. Mice and rats were euthanized with CO2 and followed with cervical dislocation or decapitation, prior to testes dissection. Rabbit tissues were purchased, while other vertebrate datasets were downloaded from the NCBI Sequencing Read Archive. This work was conducted under the approval of the Brandeis University Institutional Animal Care and Use Committee (IACUC) under the protocol #13013 to NCL.

### Library construction and deep sequencing of small RNAs and mRNAs from Drosophilid ovarium samples and Glires testes samples

To deplete the transposon-directed piRNAs which overwhelm genic piRNAs in typical total *Drosophila* ovaries small RNA libraries [13], we dissected >500 ovaries in cold PBS and that were treated to a trypsinization and gravity sedimentation protocol that ruptures most nurse cells and oocytes but leaves the layer of follicle cells intact [28]. The *D*.*mel* strain was Oregon-R, while *D*.*ere*, *D*.*yak*, and *D*.*vir* strains were the standard wild-type strains used in the genome projects and obtained from the UCSD *Drosophila* Species Stock Center. Total RNA from rodent testes was extracted using TRI-reagent (MRC) using manufacturer instructions. Small RNA in size range from 18 to 35 bases were gel purified, for *Drosophila* samples depletion from 2S rRNA was performed and libraries were constructed as described in [28].

To construct mRNAseq libraries, total RNA samples were subjected to two rounds of poly-A enrichment using biotinylated (dT)x18 oligonucleotides and the polyATtract kit (Promega). RNA-seq libraries were generated using the ScriptSeq V2 construction protocol (Epicenter, performed according to manufacturer’s instructions). Sequencing was performed on an Illumina HiSeq 2000, and 50 bases long reads were processed and split according to their index primer barcodes.

All read data that we generated for this study has been deposited in the Sequencing Reads Archive (SRA) of the Gene Expression Omnibus (GEO) under the accession number of GSE62556. All the other small RNA and mRNA datasets were downloaded from GEO and SRA, with all accession numbers recorded in S1 Table.

### Identification of genic and intergenic piRNA cluster loci expression patterns

The initial deep sequencing library preparation steps with standard processing tools is described in the Supplementary Materials Additional Experimental Procedures section. Genic piRNA clusters were determined by a process (S1c Fig) that begins with a custom script (ngs_genecentric_wig.c) that compares the counts in a WIG file to a pre-defined gene structure BED file (obtained and modified from UCSC genome browser). The script calculates the 5’UTR, exon and 3’UTR read counts for each gene in the BED file. When preforming the 5’UTR and 3’UTR counting, the script automatically extends 5’UTR or 3’UTR regions by a window of 2000bp (500bp for Drosophilids) if it detects at least 1 RPM reads within the window. After downloading original Refseq BED tables from the UCSC genome browser website, we modified the RefSeq BED files to conform to our custom script. For human, fly, and mouse, we kept the original species-specific RefSeq annotations. But for the rat and rabbit, we used the mouse RefSeq annotation first, and then supplemented it further with human RefSeq annotations where mouse annotations were missing. For the marmoset, monkey, dog, horse, pig, platypus, opossum, and chicken; we first used the human annotation, then the mouse annotation, and finally add the species specific annotations.

We curated these tables by only retaining genes with ≥10 RPMs of piRNA counts in the 3’UTR of the same strand orientation of the mRNA. In mammals, between 1–5% of genic piRNA clusters also had at least 50 RPMs of piRNAs mapping to the ORF that were kept in the analysis. We further groomed these tables by removing false positives that were genes with exceedingly high 3’UTR counts which were ambiguously attributed to a nearby transposable element, or were adjacent to another gene that was actually generating the piRNAs, was a duplicated gene name entry, or was a non-coding RNA (i.e. many mouse RefSeq non-coding RNAs with the “gm####” identifier). We finally removed additional false positives that were genes with incorrect homolog alignments caused by their simple and repetitive protein domains, such as histones, olfactory receptors and zinc finger protein genes.

Mouse, human and flies intergenic clusters lists were previously determined in [4, 5, 13, 29, 30]. Intergenic clusters for other species were detected with a custom script (ngs_coverage-nelson.c) that uses a 5 Kb sliding window, initiates a cluster when the window read count is > 1 RPM, and terminates the cluster when the read count is < 1 RPM. Intergenic piRNA cluster loci defined by contiguous intervals were then kept if they totalled ≥10RPMs. We computed the Single-stranded configuration index of piRNA clusters by first taking each cluster locus and splitting it into windows of 5 Kb each, and then for each of these windows we calculated the formula [(A-B)^2]/(A2+B2), where “A” is the plus strand read counts in RPM and “B” is the minus strand read counts in RPM within a window. The single-stranded configuration index of a cluster is the average single-stranded configuration index of all the 5Kb windows.

### Overlap analyses of gene expression profiles and genic piC loci expression profiles

The gene expression profiles for samples from human, mouse and *D*.*mel* were determined by mapping mRNA-seq reads to RefSeq transcripts with Bowtie (up to 2 mismatches). Read counts were normalized to mapping library size (RPM) and then gene length (RPKM), and finally each gene was normalized to a housekeeping gene (RpL32 in mammals and in flies, *RpL32* is also known as Rp49 in flies). Since RefSeq transcript libraries are deficient in other animals, for the other Drosophilids, mapped reads were then transformed to *D*.*mel* gene models through orthologs listed in the OrthoDB database and then RPM counts were determined as described in [75]. mRNA-seq reads for non-human Primates, Laurasiatherians, and other tetrapods were mapped to their respective genomes, and then the counting algorithm from genic piC discovery was applied to yield exonic counts that were as accurate as mapping to RefSeq transcripts, except that no additional curation was applied.

Differential gene expression analysis between Asb1 KO and Het mutant mouse tissues was conducted with the edgeR package [76] on two biological replicates from testes and one sample of kidney. For predicting targets of piC-Asb1, Asb1 piRNAs were queried against the mouse RefSeq transcripts using the “blastn-short” command in BLAST [77] and then sorting for BLAST results with an e-value≤1, which in this query frequently demanded at least 13bp of complementarity between the piRNA and the predicted target. Custom Perl scripts and SQL queries counted the BLAST matches and gene expression changes.

For the species comparisons Venn diagrams, the genic piC names and the gene expression profiles were rooted to the best annotated model organism gene names from *D*.*mel* for Drosophilids; and human and mouse for mammals and chicken. Genes and genic piC loci could thus be compared between species with SQL queries. Overlaps analyses in genic piC locus conservation required threshold piRNA expressions to be ≥10 RPMs (Figs 1F and 2G) and ≥20 RPMs (S2B and S4G Figs). Overlap in gene expression profiles were defined as similar expression levels of RPKM values normalized against the RpL32/Rp49 housekeeping gene to be within 3 fold of each two species comparison (Log10 delta value ≤ 0.5) and within 2 fold for three species comparisons (Log10 delta value standard deviation ≤ 0.3) for analyses in Figs 1F and 2G. For gene expression analyses in S2B and S4G Figs, the Log10 delta value ≤0.3 and Log10 delta value standard deviation ≤ 0.177 was used. To test if the distribution of piC locus overlaps were significantly different from the distribution of similar gene expression profiles, we calculated both the Chi-square test and the Ratios proportion Z-score test with Bonferroni correction. The human Study #2 dataset is a second independent sample of small RNAs generated from human testes, and it shares much similarity with the human Study #1 dataset, such as similar piRNA cluster loci patterns in Figs 2B, 2C and S4I. All human piC loci comparisons in Fig 3 were done with the human Study#1 datasets after we had confirmed highly similar profiles and counts for all ECpiC loci in the human Study#2 dataset.

### Evolution and feature analyses of genic piRNA cluster loci

We ranked genic piC loci by piRNA abundance (RPMs) and selected top cohorts of 278 and 332 genic piC loci for fly and mouse, respectively. These sets were divided into a top and bottom half, and compared to lists of negative control genes that do not generate piRNAs. The negative control list contained a total of 3 times as many genes as genic piC loci to diminish selection bias, and >90% of these negative control genes were also expressed in both fly follicle cell enriched samples and mouse adult testes. The 3’UTR boundaries of the genic piC loci were defined by the previous piRNA tracking algorithm while the negative controls were selected based on the longest Refseq-annotated 3’UTR. In order to ensure that genes in negative control set have approximately similar 3’UTR length compared to genic piC loci set, we padded the 3’UTR in fly by 250 to 500 bases and in mouse by 500 to 1100 bases, unless the extension overlaps with a gene in the same strand. The genomic coordinates for the 3’UTRs and CDS (minus introns) were determined for each negative control gene and genic piC; and used to track and count predicted RNA structural elements and phastCons and phyloP scores.

RNA structural elements from fly genome (Release 5/Dm3) were determined with RNAz, Evofold, and REAPR applied to the 12 Drosophilids alignments with the deviation parameter, dev = 20 and confidence scores >0.6 [39–41], while only REAPR was applied to the mouse genome (Mm10) using an alignment of 8 Glire genomes and measured at deviation levels of dev = 10 and confidence scores >0.8. The per-base values of phastCons [43] and phyloP [44] scores were downloaded from UCSC Genome browser with the exception of fly phyloP scores computed by David Garfield at the EMBL, and were based off the 12 Drosophilids genomes and 60 vertebrate genomes alignments. Total per-base values were summed and then averaged by the base length for each gene using BEDtools [78] and custom Perl scripts to count the features for each gene. Sequence motif analysis was performed with MEME [42] using an “OOPS (only once per sequence)” model; and with GLAM2 on CDS and 3’UTR sequences of the longest isoform from the masked genome.

To determine the gain and loss rates for genic piC loci in Drosophilids and tetrapods, we constructed phylogenetic trees according to the Gregorian clock of millions of years of divergence and the tabulated expression or absence of expression for each piC loci with gene orthologs present between all the species. We followed the similar procedure of measuring gain and loss rates of miRNA genes as detailed in [20], using the COUNTS program [79] and the Wagner parsimony approach. Additional experimental procedures are in the Supplementary Materials document describing the verification of gene expression using RT-qPCR, small RNA northern blotting, and the PIWI IP from OSS cells and PIWIL2 IP from Glire testes.

## Supporting Information

S1 Text Supplemental text discussion and additional methods. (DOCX)

S1 Fig Discovery process of genic piRNA clusters (piC) loci in flies and mammals. (**A**) Applying the DIOPT program [80] predicts 403 genic piC loci putatively conserved between *D*.*mel* and mouse, but the confidence in determining whether these genes are true orthologs is hampered by the >600 Million Years of evolution between species and gene family expansions in mice. (**B**) Flowchart of the piC locus discovery algorithm that combines an automatic output of candidate genic piC loci which are then curated into confident lists for each small RNA library. (**C**) *D*.*mel* genic piC loci called out our deep sequencing and algorithm. (Left) Genome browser snapshots of three top genic piC loci with purple arrows point to bulk of piRNAs. (Middle) Pie charts of the 5' nucleotide base compositions of the piRNAs from these three genic piC loci. (Right) Length distribution of reads from these genic piC loci and the entire D.mel library. (**D**) The 5' nucleotide base compositions of all the small RNAs from each vertebrate adult testes library analyzed in this study. The length distributions and the 5' nucleotide base composition of small RNA reads from selected genic piC loci for (**E**) mouse and (**F**) human adult testes.
(TIF)

S2 Fig Additional analyses of Drosophilids genic piC loci expression and PIWI-pathway associated gene expression. (**A**) Euler diagram of genic piC loci detected in each Drosophilid ovarium sample. Since there were so few genic piC loci discovered in *D*.*yak*, we omitted this specie from the comparison in Fig 1F. (**B**) Venn diagrams comparing the overlap of genic piC loci and gene expression profiles from Drosophilids follicle samples using piRNA and expression cutoffs that are twice as stringent as in Fig 1F. (**C**) Northern blots probing against genic piRNAs, TE-directed *roo* piRNAs, and microRNA-12 and the U6 spliceosomal RNA as loading/probing controls. Total RNA from OSS cells and highly concentrated bulk small RNAs from Drosophilid abdomens were used in this experiment. The pervasive but low piRNA signals for genic piRNAs across all the Drosophilid sample lanes are due to probe cross-hybridization seen in two different experiments. (**D**) Genes identified by mass spectrometry that are associated with PIWI from an IP in OSS cells which robustly express genic piC loci. Next to this is a heat map of their strong expression profiles in all ovarium samples of the four Drosophilids. (**E**) Heatmap of the top 100 genic piC loci ranked by piRNA expression in *D*.*mel*. (**F**) Estimation of the gain and loss of genic piC loci along each branch of the phylogeny for four Drosophilids, with the effective gain rates shown in red.
(TIF)

S3 Fig Evolutionary patterns of the two major *D*.*mel* intergenic piC loci, piC-*42AB* and *flam*. (**A**) Genome browser snapshots for multiple Drosophilid genomes across the *DIP1-CG14621* syntenic region that demarcates a *flam-*like piC locus. Purple arrows point to the start of bulk of piRNAs. Plus strand read peaks are red, minus strand read peaks are blue. Double-hashed lines mark a large break the region that disrupts the gene synteny. Beyond D.ere and D.yak, There is a lack of a large TE-rich segment separating *DIP1-CG14621* in the other species. (**B**) Snapshots across the *Pld-Jing* syntenic region that demarcate a *42AB*-like piC locus in *D*.*mel* and its closest relatives *D*.*sec* and *D*.*sim*, which display a TE-rich region. There is a lack of a large TE-rich segment separating *Pld*-*Jing* in the other species.
(TIF)

S4 Fig Additional analyses of mammalian genic piC expression and evolutionary patterns. (**A**) Northern blots confirming species-specific genic piC expression from Fig 2D–2F. (**B**) Diagram of the developmental stages in mammalian spermatogenesis explaining the differential expression patterns of genic and intergenic piRNAs. The rationale for sequencing PIWIL2 IPs from adult testes is to capture additional genic piC loci. (**C**) Read length distributions of the 10dpp testes and PIWIL2 IP small RNA libraries from marmoset, mouse, rat and rabbit, and compared to the adult testes total small RNAs in other primates and mouse and rabbit. (**D**) Genome browser snapshots of piRNA clusters conserved in Glires and which exhibit stronger piRNA expression in 10dpp testes, piC-*Abl2* (left); and piC-*Elk4* (right). Purple arrows point to the start of bulk of piRNAs. Plus strand read peaks are red, minus strand read peaks are blue. **(E-I)**Venn diagrams the number of genic piC loci from mouse, rat and rabbit testes in each of the different stages and samples (**E**), and comparing between the species for a given stage or sample (**F**). Overlapping areas are genic piC loci shared between the two libraries. (**G**) The overlap of Glire species genic piC loci in all samples, between human and mouse, and between Primates and Glires. (**H**) Overlap of genic piC loci and gene expression profiles from adult testes from human, mouse and dog, using piRNA and expression cutoffs that are twice as stringent as in Fig 2G, right-most set of diagrams. (**I**) Overlap of identical genic piC loci called by our pipeline from two independently-constructed small RNA libraries from human and chicken adult testes. (**J**) Glires-focused heatmap of the top 100 genic piC loci ranked by piRNA expression in mouse. (**K**) Primates-focused heatmap of the top 100 genic piC loci ranked by piRNA expression in human. (**L**) Estimation of the gain and loss of genic piC loci along each branch of the phylogeny between eleven mammals and the chicken, with the effective gain rates shown in red.
(TIF)

S5 Fig Evolution and feature analyses of fly and mouse genic piC loci. (**A**) Graph displaying similar numbers of genic piC loci and similar average 3’UTR lengths in between the genic piC loci and the negative control genes for the fly and mouse sets, which do not generate piRNAs. Comparison of the average number of predicted conserved structural elements residing in genic piC transcripts versus transcripts that do not generate piRNAs for fly genes (**B**) and mouse genes (**C**). Set#1 is the top half of the genic piC loci with the highest piRNA counts, Set#2 is the bottom half of the genic piC loci with lower piRNA counts. Asterisks mark *p<0*.*01*, T-test. Summary of top scoring sequence motifs on fly genes (**D**) and mouse genes (**E**) from MEME and GLAM2 analyses. The MEME output scores report the number of transcripts out of the total query containing the top motif and the associated *p*-value, while the GLAM2 scores reflect the reproducible discovery of the motif during iteration steps in the algorithm. Violin plots of normalized phastCons and phyloP scores for each gene segment (ORF versus 3’UTR) being compared between genic piC loci and negative control genes for fly (**F**) and mouse (**G**). Set#1 is the top half of the genic piC loci with the highest piRNA counts, Set#2 is the bottom half of the genic piC loci with lower piRNA counts. Asterisks mark *p<0*.*05*, Mann-Whitney test. ECpiC loci Eutherian-Conserved piRNA clusters, LCpiC loci, Less-Conserved piRNA clusters.
(TIF)

S6 Fig Full display of genome browser snapshots of ECpiC loci from Fig 3C–3E. The full compilation across 11 mammals and the chicken of two intergenic ECpiC loci shown in (**A**) and (**B**), and three genic ECpiC loci shown in (**C**), (**D**) and (**E**). The majority of piRNAs from intergenic ECpiC #18 are actually antisense to the protein-coding transcript of the STOX1 gene. Plus strand read peaks are red, minus strand read peaks are blue. Purple arrows point to the start of bulk of piRNAs.
(TIF)

S1 Table Statistics and accession numbers for Illumina libraries of small RNAs and mRNAs generated and analyzed in this study. Shaded lines are small RNA libraries. Accessions and library citations are listed on the right most columns and at the bottom of the table.
(PDF)

S2 Table Multi-tab curated lists of the genic piC loci across the animal species determined in this study. (XLSX)

S3 Table Conservation intergenic piC loci expression as best defined in human and mouse and segregated according to Eutherian-Conserved piRNA clusters (ECpiC) loci and Less-Conserved piC (LCpiC) loci. (XLSX)

S4 Table Mouse genes up-regulated in the Asb1 mutant KO testis and have predicted base-pairing Asb1-piRNAs (Refers to Fig 5H). (XLSX)

S5 Table Oligonucleotides used in this study. (PDF)
