# Supplementary material for: Conserved piRNA Expression from a Distinct Set of piRNA Cluster Loci in Eutherian Mammals
Source: PLoS Genet. 2015 Nov 20;11(11):e1005652. doi: 10.1371/journal.pgen.1005652 (PMC4654475; doi:10.1371/journal.pgen.1005652)
Supplement: S1 Text — (DOCX) [file pgen.1005652.s001.docx]

**SUPPLEMENTARY TEXT DISCUSSION**

Parts of our study depend upon piRNA datasets generated by other labs that are outside our quality control procedures for library construction and sequencing. However, we purposefully chose to analyze only the available piRNA dataset that displayed sufficient depth and quality, and bypassed other existing Drosophilids and mammalian piRNA libraries because they may have lacked enough sequencing depth, were not diverse enough in covering all piRNA repertoires, or were already covered by our own library sequencing efforts [1-5]. For example, in contrast to the highly diverse population of piRNAs in the *D.mel* ovary, *D.mel* testes piRNAs were not diverse and mainly restricted to one cluster on the X chromosome and the *Su(Ste)* clusters littered in unassembled heterochromatin [2]. This lack of diversity in *D.mel* male-specific piC loci might extend to other Drosophilids and supports our rationale to mainly focus on ovarium samples. Mammalian ovaries are also technically challenging to harvest in large amounts, but in mouse oocytes, endo-siRNAs appear to dominate over piRNAs [5-7]. This contrasts with fish and frog oocytes that are highly abundant in material and piRNAs [8, 9], yet are evolutionarily more distant to mammals than the chicken.

We considered whether some genic piC loci might only be detected in particular earlier developmental stages in gametogenesis and embryogenesis. We compared the genic piC loci in mouse and rat between the adult testes and 10dpp testes, a stage where we and others have previously shown was more amenable to genic piC detection because post-pachytene intergenic piRNAs were not yet expressed (S4B Fig)[10-12]. Since our antibody to mouse PIWIL2 cross-reacted with rat and rabbit PIWIL2, we also hypothesized that we could increase the coverage of genic piC loci from adult testes by sequencing piRNAs from PIWIL2 IP loci (S4C–E Fig). In the rat and rabbit, there were many distinct genic piC loci detected only in the 10dpp testes and the PIWIL2 IP compared to the adult testes. Genic piC loci in the rat are also subdued in the adult from extensive intergenic piRNA expression, so profiling 10dpp testes and PIWIL2 IP loci can compensate for the overshadowing of genic piC loci in certain mammalian adult testes small RNA datasets. For the mouse, the 10dpp testes piRNA dataset was very comprehensive, covering nearly all the genic piRNAs detected in adult testes and PIWIL2 IP loci (S4E Fig).

When comparing the genic piC loci between Glire species within these special samples, there was ample overlap between mouse and rat 10dpp testes piRNAs (S4E Fig, left), but surprisingly much less overlap of genic piC loci in the PIWIL2 IPs between mouse, rat, and rabbit, (S4E Fig, middle) which perhaps reflect the more promiscuous transcriptome diversity in adult testes as seen by other groups [13, 14]. When considering in total all the genic piC loci from all stages between Glire species, there were still many species-specific genic piC loci (S4F Fig, left) that we believe will likely represent the diversity of genic piC loci in other developmental stages of other mammalian testes.

It is possible that additional small RNA libraries from other developmental stages and IP loci could improve our piRNA profiling approach. However, it is also logistically challenging to obtain testes from most other mammals besides Glires. Obtaining pre-pubescent testes from non-Glire mammals would be even more challenging, although if adult testes tissues were available and suitable for protein extract creation, PIWIL2 IP loci might increase our depth of genic piC coverage. Deeper sequencing of the adult gonadal small RNA libraries in our study in theory might yield more diversity of piRNA reads to capture more genic piC loci. However, deeper sequencing would also hit diminishing returns because more intergenic piRNAs will also be sequenced, and our analyses normalize piRNA read counts to RPM values.

Deeper sequencing also cannot circumvent the issue that some animal genome assemblies are still incomplete drafts that can also partly impede our detection of certain piC loci. Genome assembly gaps and fragmented contigs that make it difficult to determine synteny has slightly hampered our determination of piC gene orthologs in opossum, platypus and chicken (Fig 4A). We were also unable to pinpoint the two major intergenic piC loci *42AB* and *flam* in *D.sec* because of fragmented contigs (S3Fig). Even the human, mouse, and *D.mel* genome sequences that are the most complete of genome projects still contain some gaps in heterochromatin segments, but these gaps are more likely to only impact the characterization of intergenic piC loci and TE-directed piRNAs.

**ADDITIONAL METHODS**

**Initial deep sequencing library processing steps**

Our deep-sequencing processing pipeline is called from the shell script “process.c”. The pipeline first checks read quality by FastQC [15], sorted according to the barcode sequence in their 5' adaptor, and then adaptor sequences were trimmed by FASTX-Toolkit [16]. Reads were mapped to the various reference genome listed in Table S1 by using Bowtie[17] (allowing maximum 2 mismatches). Structural RNAs, viral small RNAs, and miRNAs were initially filtered for a subset of the small RNA libraries by cross-mapping to a custom database, but this step was later omitted from other mammals because the contribution of these other RNAs was deemed negligible to the overall analyses on genic and intergenic piRNAs. Read counts were normalized by the total number of reads mapped to genome (RPM) and were converted to SAM, BAM, BED and WIG formats using SAMtools [18] and BEDtools [19].

All accession numbers recorded in S1 Table represent libraries coming from the following references: [11, 13, 14, 20-33]

**Verification of gene expression using RT-qPCR.**

To experimentally validate RNA expression changes, reverse transcription was performed using 0.2 µg of RNA and M-MLV reverse transcriptase (Promega). cDNA synthesis was performed by random primers and qPCR was performed using GoTaq SybrGreen Master mix (Promega) on Bio-Rad C1000 machine. Oligonucleotides used for specific sequences amplification are listed in Table S4. Relative changes in gene expression were calculated using the 2^^ΔΔCt^ method [34]. *Rp49* (also called *RpL32*) mRNA was used for normalization in fly samples.

**Small RNA northern blotting.**

Total small RNAs from flies were obtained from 2 g of flies that were snap-frozen in liquid nitrogen and abdomens were separated with sieves. Abdomens were pulverized and total RNA was extracted. 5 mg of total RNA was loaded on the 2% low melting point agarose gel and separation was done by native electrophoresis in 1xTBE buffer. Gel region containing small RNAs in the size range 15-40 bases was excised and RNA was eluted overnight with elution buffer and phenol, then back extracted with chloroform and precipitated with isopropanol. Complete total RNA from mouse, rat and rabbit testes was from pulverized frozen testes extracted in Tri-reagent. 30ug of total glires testes RNA or 10 mg of fly enriched small RNA fraction was loaded on the 15% Urea-polyacrylamide gel; and small RNA northern was done essentially as in [35]; piRNAs and small RNA of interest were detected using specific oligo probes (see S5 Table).

**PIWI IP from OSS cells and PIWIL2 IP from Glire testes.**

Protein A/G magnetic beads (Pierce) were coated with anti-PIWI antibodies at 60 µg per 60 µl of beads for 2 hours at 23°C, washed with PBS and resuspended in initial volume of Q buffer (20mM Hepes-KOH pH 7.9; 10% glycerol; 0.1 M KOAc; 0.2 mM EDTA; 1.5 mM MgCl_2_; 0.5 mM DTT; 1X Roche Complete EDTA-free Protease Inhibitor Cocktail; 0.5% NP40). Cells were lysed in Q-column binding buffer (5x volume in relation to the dry pellet), with 50 strokes of dounce homogenizer and sonicated 2 rounds for 30 sec (with 2 sec pulse/2 sec pause cycle, Qsonica); centrifuged 15000 rpm 30min 4°C. For isolating the cytoplasmic fraction cells were lysed in hypotonic buffer (15 mM HEPES-KOH at pH 7.6, 10 mM KCl, 5 mM MgOAc, 3 mM CaCl_2_, 300 mM sucrose, 0.1% Triton X-100, 1 mM DTT, 1× Complete protease inhibitors [Roche]) by douncing with 30 strokes of dounce homogenizer with tight pestle. The lysate was spun for 10 min at 2000 g; the supernatant (cytoplasmic fraction) was cleared by centrifugation at 15,000g for 30 min. IPs were performed by incubating with PIWI antibody coated beads on rotating wheel for 1.5 hours at 4°C. Beads were washed 5 times with Q buffer and elution was done using Laemmlie SDS-PAGE loading buffer for 10 min at 23°C followed by cooking for 5 min at 95°C. The elution fraction was then loaded on the Bis-Tris Nu-PAGE gel 4-12% (Invitrogen) with following imperial blue staining. The bands were cut; extraction and proteomics analysis were performed at Taplin Mass Spectrometry facility (Harvard Medical School).

Adult testes from mice, rats and rabbits were decapsulated and converted into lysates using the same conditions above for making lysates from OSS cells. A rabbit polyclonal antibody was raised against the mouse PIWIL2 protein sequence RGLSANLVRKDREE (aa:100-113), and this affinity purified antibody cross-reacts with rat and rabbit PIWIL2 in IPs (S4L Fig). 50ug of this antibody was bound to 100 ul of Protein A/G magnetic beads and incubated for 2 hours at 4°C with 2mL of mouse, rat or rabbit testes extract. Beads were washed 5 times with Q buffer and RNAs were extracted with Trizol and then subjected to small RNA library construction. Although some PIWIL1 carryover was likely in the PIWIL2 IPs from rat and rabbit testes lysates, the samples were still sufficient in quality for this analysis to examine our breadth of piRNA coverage in S4 Fig, and did not affect the analysis in Fig 2 and Fig 3.

**SUPPLEMENTARY TEXT REFERENCES**

1. Malone CD, Brennecke J, Dus M, Stark A, McCombie WR, Sachidanandam R, et al. Specialized piRNA pathways act in germline and somatic tissues of the Drosophila ovary. Cell. 2009;137(3):522-35. Epub 2009/04/28.

2. Nishida KM, Saito K, Mori T, Kawamura Y, Nagami-Okada T, Inagaki S, et al. Gene silencing mechanisms mediated by Aubergine piRNA complexes in Drosophila male gonad. Rna. 2007;13(11):1911-22. Epub 2007/09/18.

3. Rozhkov NV, Aravin AA, Zelentsova ES, Schostak NG, Sachidanandam R, McCombie WR, et al. Small RNA-based silencing strategies for transposons in the process of invading Drosophila species. Rna. 2010;16(8):1634-45. Epub 2010/06/29.

4. Murchison EP, Kheradpour P, Sachidanandam R, Smith C, Hodges E, Xuan Z, et al. Conservation of small RNA pathways in platypus. Genome research. 2008;18(6):995-1004. Epub 2008/05/09.

5. Tam OH, Aravin AA, Stein P, Girard A, Murchison EP, Cheloufi S, et al. Pseudogene-derived small interfering RNAs regulate gene expression in mouse oocytes. Nature. 2008;453(7194):534-8. Epub 2008/04/12.

6. Flemr M, Malik R, Franke V, Nejepinska J, Sedlacek R, Vlahovicek K, et al. A retrotransposon-driven dicer isoform directs endogenous small interfering RNA production in mouse oocytes. Cell. 2013;155(4):807-16.

7. Watanabe T, Totoki Y, Toyoda A, Kaneda M, Kuramochi-Miyagawa S, Obata Y, et al. Endogenous siRNAs from naturally formed dsRNAs regulate transcripts in mouse oocytes. Nature. 2008;453(7194):539-43. Epub 2008/04/12.

8. Houwing S, Kamminga LM, Berezikov E, Cronembold D, Girard A, van den Elst H, et al. A Role for Piwi and piRNAs in Germ Cell Maintenance and Transposon Silencing in Zebrafish. Cell. 2007;129(1):69-82.

9. Lau NC, Ohsumi T, Borowsky M, Kingston RE, Blower MD. Systematic and single cell analysis of Xenopus Piwi-interacting RNAs and Xiwi. The EMBO journal. 2009;28(19):2945-58. Epub 2009/08/29.

10. Aravin AA, Sachidanandam R, Girard A, Fejes-Toth K, Hannon GJ. Developmentally regulated piRNA clusters implicate MILI in transposon control. Science. 2007;316(5825):744-7.

11. Li XZ, Roy CK, Dong X, Bolcun-Filas E, Wang J, Han BW, et al. An ancient transcription factor initiates the burst of piRNA production during early meiosis in mouse testes. Molecular cell. 2013;50(1):67-81.

12. Robine N, Lau NC, Balla S, Jin Z, Okamura K, Kuramochi-Miyagawa S, et al. A broadly conserved pathway generates 3' UTR-directed primary piRNAs. Current Biology. 2009;19(22). Epub 2009/12/17.

13. Brawand D, Soumillon M, Necsulea A, Julien P, Csardi G, Harrigan P, et al. The evolution of gene expression levels in mammalian organs. Nature. 2011;478(7369):343-8.

14. Necsulea A, Soumillon M, Warnefors M, Liechti A, Daish T, Zeller U, et al. The evolution of lncRNA repertoires and expression patterns in tetrapods. Nature. 2014;505(7485):635-40.

15. Andrews S. FastQC. <http://http://www.bioinformatics.babraham.ac.uk/projects/fastqc/2010>.

16. Hannon-Lab. FASTX-Toolkit: FASTQ/A short-reads pre-processing tools. <http://hannonlab.cshl.edu/fastx_toolkit/2009>.

17. Langmead B. Aligning short sequencing reads with Bowtie. Curr Protoc Bioinformatics. 2010;Chapter 11:Unit 11 7.

18. Li H, Handsaker B, Wysoker A, Fennell T, Ruan J, Homer N, et al. The Sequence Alignment/Map format and SAMtools. Bioinformatics. 2009;25(16):2078-9.

19. Quinlan AR, Hall IM. BEDTools: a flexible suite of utilities for comparing genomic features. Bioinformatics. 2010;26(6):841-2.

20. Coleman SJ, Zeng Z, Hestand MS, Liu J, Macleod JN. Analysis of unannotated equine transcripts identified by mRNA sequencing. PloS one. 2013;8(7):e70125.

21. Derti A, Garrett-Engele P, Macisaac KD, Stevens RC, Sriram S, Chen R, et al. A quantitative atlas of polyadenylation in five mammals. Genome research. 2012;22(6):1173-83.

22. Ha H, Song J, Wang S, Kapusta A, Feschotte C, Chen KC, et al. A comprehensive analysis of piRNAs from adult human testis and their relationship with genes and mobile elements. BMC genomics. 2014;15:545.

23. Hirano T, Iwasaki YW, Lin ZY, Imamura M, Seki NM, Sasaki E, et al. Small RNA profiling and characterization of piRNA clusters in the adult testes of the common marmoset, a model primate. Rna. 2014;20(8):1223-37.

24. Lian C, Sun B, Niu S, Yang R, Liu B, Lu C, et al. A comparative profile of the microRNA transcriptome in immature and mature porcine testes using Solexa deep sequencing. The FEBS journal. 2012;279(6):964-75.

25. Margolin G, Khil PP, Kim J, Bellani MA, Camerini-Otero RD. Integrated transcriptome analysis of mouse spermatogenesis. BMC genomics. 2014;15:39.

26. Meunier J, Lemoine F, Soumillon M, Liechti A, Weier M, Guschanski K, et al. Birth and expression evolution of mammalian microRNA genes. Genome research. 2013;23(1):34-45.

27. Platt RN, 2nd, Vandewege MW, Kern C, Schmidt CJ, Hoffmann FG, Ray DA. Large numbers of novel miRNAs originate from DNA transposons and are coincident with a large species radiation in bats. Molecular biology and evolution. 2014;31(6):1536-45.

28. Robine N, Lau NC, Balla S, Jin Z, Okamura K, Kuramochi-Miyagawa S, et al. A broadly conserved pathway generates 3' UTR-directed primary piRNAs. Current Biology. 2009;19(24):2066-76. Epub 2009/12/17.

29. Yan Z, Hu HY, Jiang X, Maierhofer V, Neb E, He L, et al. Widespread expression of piRNA-like molecules in somatic tissues. Nucleic acids research. 2011;39(15):6596-607.

30. Yang Q, Hua J, Wang L, Xu B, Zhang H, Ye N, et al. MicroRNA and piRNA profiles in normal human testis detected by next generation sequencing. PloS one. 2013;8(6):e66809.

31. Yu Y, Fuscoe JC, Zhao C, Guo C, Jia M, Qing T, et al. A rat RNA-Seq transcriptomic BodyMap across 11 organs and 4 developmental stages. Nature communications. 2014;5:3230.

32. Gunawan A, Sahadevan S, Neuhoff C, Grosse-Brinkhaus C, Gad A, Frieden L, et al. RNA deep sequencing reveals novel candidate genes and polymorphisms in boar testis and liver tissues with divergent androstenone levels. PloS one. 2013;8(5):e63259.

33. Hoeppner MP, Lundquist A, Pirun M, Meadows JR, Zamani N, Johnson J, et al. An improved canine genome and a comprehensive catalogue of coding genes and non-coding transcripts. PloS one. 2014;9(3):e91172.

34. Winer J, Jung CK, Shackel I, Williams PM. Development and validation of real-time quantitative reverse transcriptase-polymerase chain reaction for monitoring gene expression in cardiac myocytes in vitro. Anal Biochem. 1999;270(1):41-9.

35. Lau NC. Analysis of small endogenous RNAs. Curr Protoc Mol Biol. 2008;Chapter 26:Unit26 7. Epub 2008/01/31.
