# Supplementary figures and images for: Conserved piRNA Expression from a Distinct Set of piRNA Cluster Loci in Eutherian Mammals
Source: PLoS Genet. 2015 Nov 20;11(11):e1005652. doi: 10.1371/journal.pgen.1005652 (PMC4654475; doi:10.1371/journal.pgen.1005652)

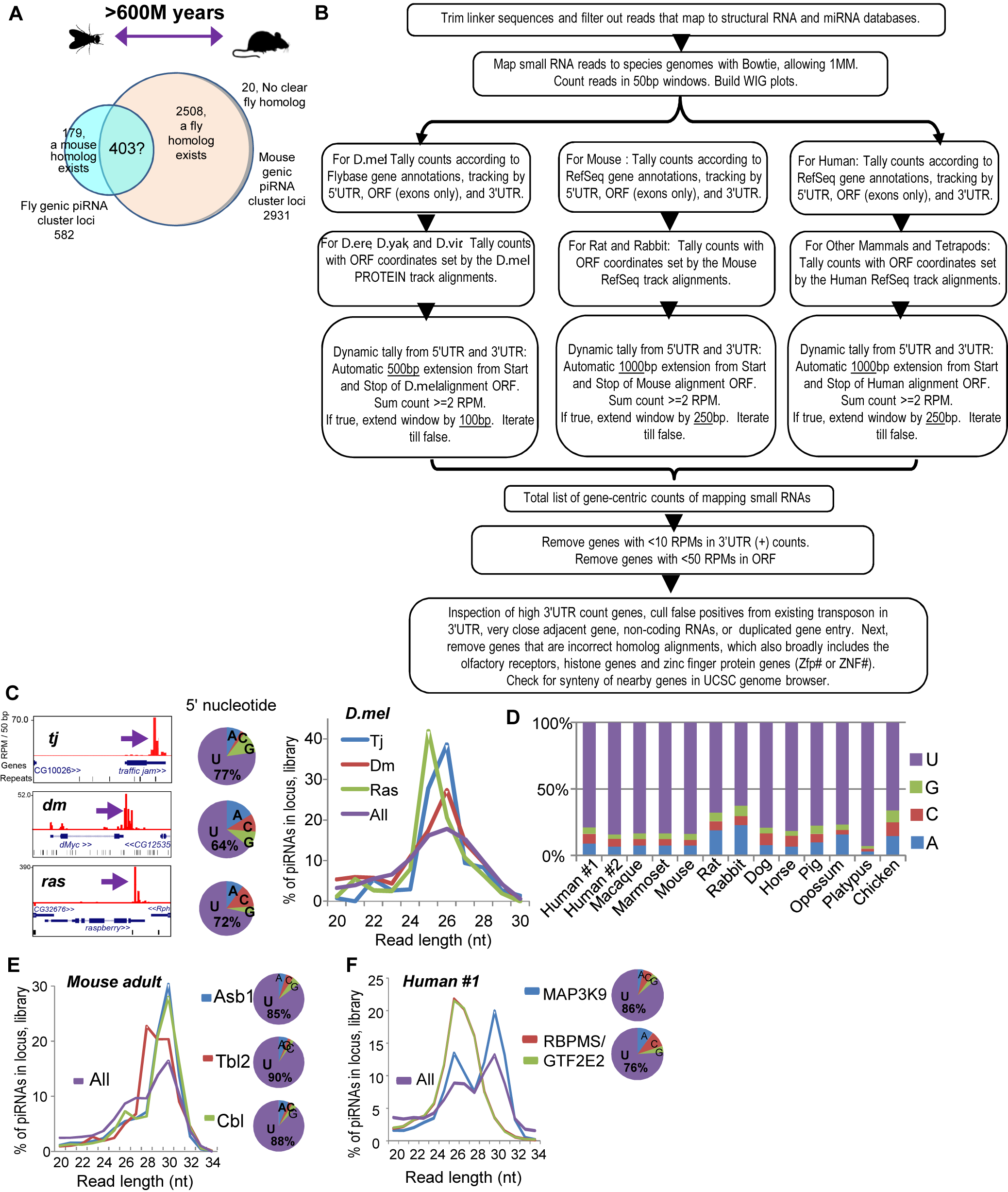

Supplement: S1 Fig — (A) Applying the DIOPT program [80] predicts 403 genic piC loci putatively conserved between D.mel and mouse, but the confidence in determining whether these genes are true orthologs is hampered by the >600 Million Years of evolution between species and gene family expansions in mice. (B) Flowchart of the piC locus discovery algorithm that combines an automatic output of candidate genic piC loci which are then curated into confident lists for each small RNA library. (C) D.mel genic piC loci called out our deep sequencing and algorithm. (Left) Genome browser snapshots of three top genic piC loci with purple arrows point to bulk of piRNAs. (Middle) Pie charts of the 5' nucleotide base compositions of the piRNAs from these three genic piC loci. (Right) Length distribution of reads from these genic piC loci and the entire D.mel library. (D) The 5' nucleotide base compositions of all the small RNAs from each vertebrate adult testes library analyzed in this study. The length distributions and the 5' nucleotide base composition of small RNA reads from selected genic piC loci for (E) mouse and (F) human adult testes. (TIF) [file pgen.1005652.s002.tif]

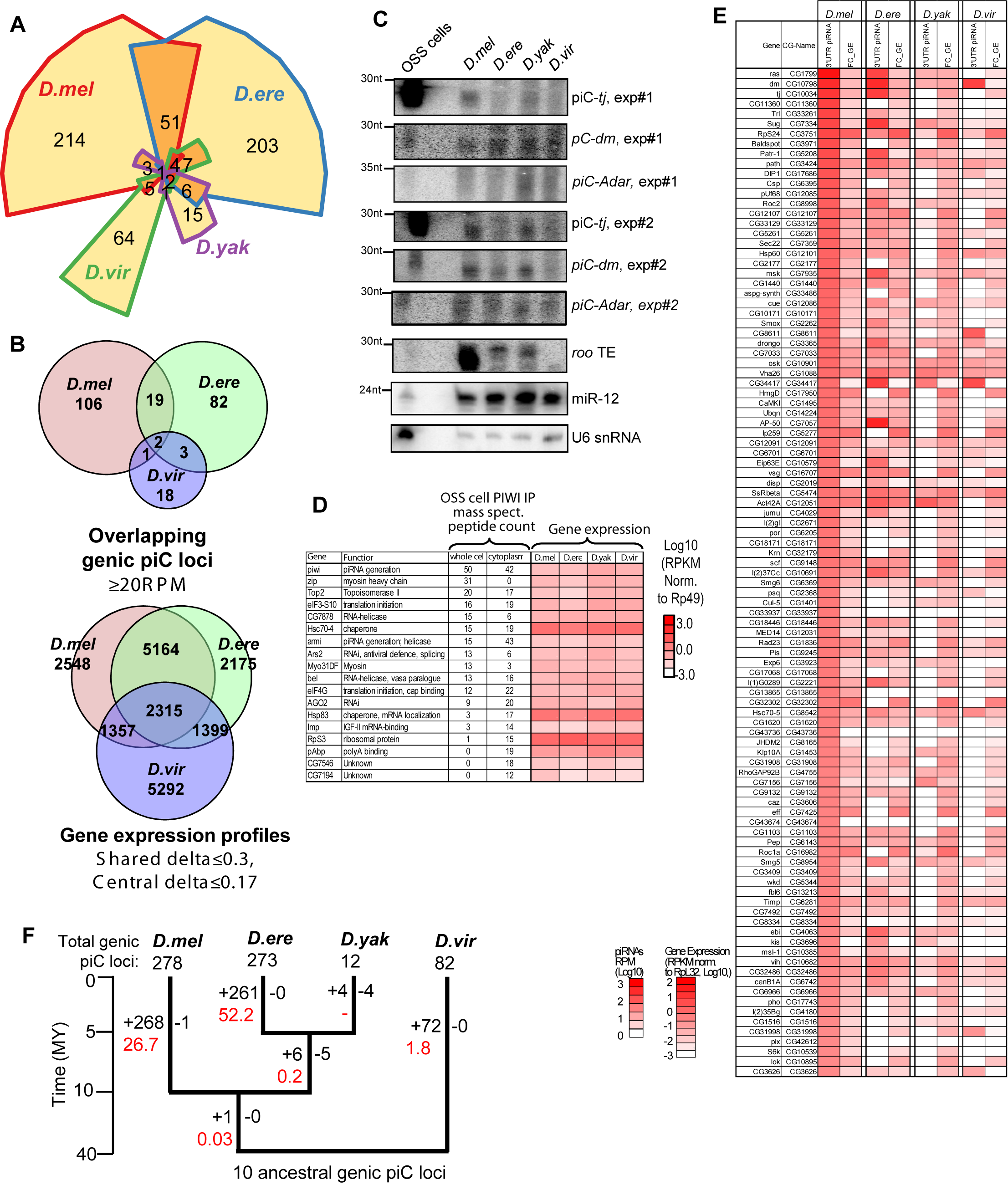

Supplement: S2 Fig — (A) Euler diagram of genic piC loci detected in each Drosophilid ovarium sample. Since there were so few genic piC loci discovered in D.yak, we omitted this specie from the comparison in Fig 1F. (B) Venn diagrams comparing the overlap of genic piC loci and gene expression profiles from Drosophilids follicle samples using piRNA and expression cutoffs that are twice as stringent as in Fig 1F. (C) Northern blots probing against genic piRNAs, TE-directed roo piRNAs, and microRNA-12 and the U6 spliceosomal RNA as loading/probing controls. Total RNA from OSS cells and highly concentrated bulk small RNAs from Drosophilid abdomens were used in this experiment. The pervasive but low piRNA signals for genic piRNAs across all the Drosophilid sample lanes are due to probe cross-hybridization seen in two different experiments. (D) Genes identified by mass spectrometry that are associated with PIWI from an IP in OSS cells which robustly express genic piC loci. Next to this is a heat map of their strong expression profiles in all ovarium samples of the four Drosophilids. (E) Heatmap of the top 100 genic piC loci ranked by piRNA expression in D.mel. (F) Estimation of the gain and loss of genic piC loci along each branch of the phylogeny for four Drosophilids, with the effective gain rates shown in red. (TIF) [file pgen.1005652.s003.tif]

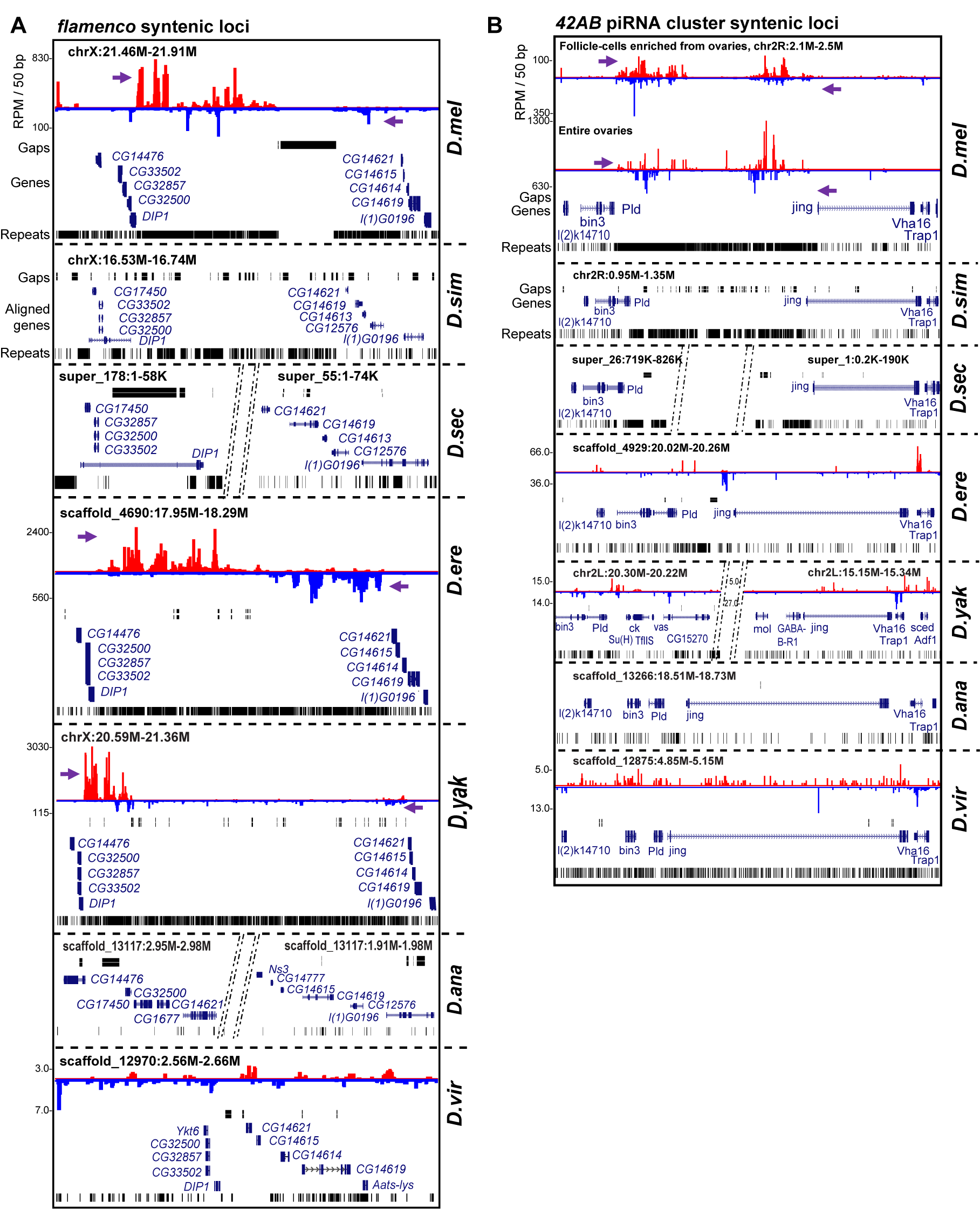

Supplement: S3 Fig — (A) Genome browser snapshots for multiple Drosophilid genomes across the DIP1-CG14621 syntenic region that demarcates a flam-like piC locus. Purple arrows point to the start of bulk of piRNAs. Plus strand read peaks are red, minus strand read peaks are blue. Double-hashed lines mark a large break the region that disrupts the gene synteny. Beyond D.ere and D.yak, There is a lack of a large TE-rich segment separating DIP1-CG14621 in the other species. (B) Snapshots across the Pld-Jing syntenic region that demarcate a 42AB-like piC locus in D.mel and its closest relatives D.sec and D.sim, which display a TE-rich region. There is a lack of a large TE-rich segment separating Pld-Jing in the other species. (TIF) [file pgen.1005652.s004.tif]

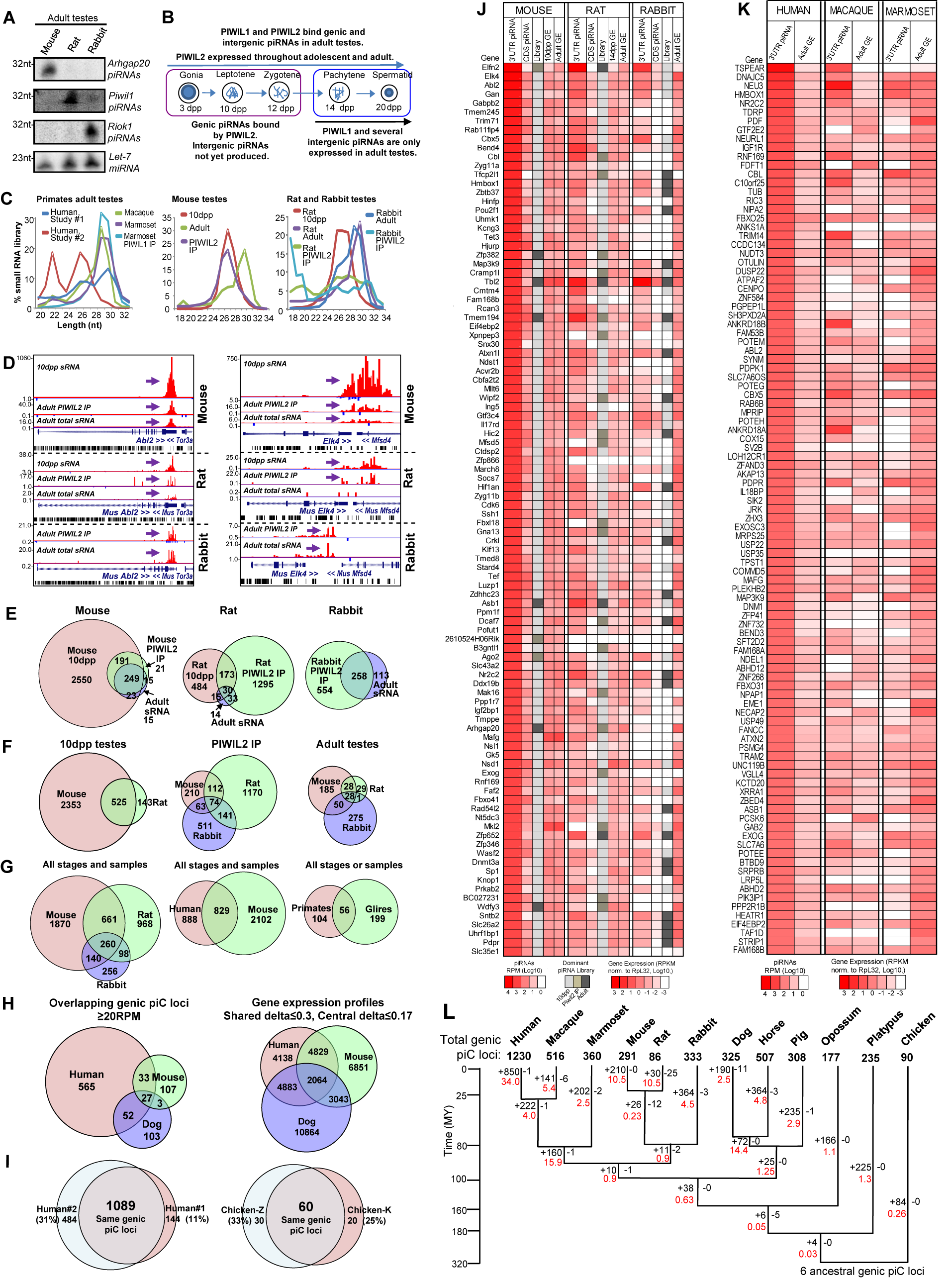

Supplement: S4 Fig — (A) Northern blots confirming species-specific genic piC expression from Fig 2D–2F. (B) Diagram of the developmental stages in mammalian spermatogenesis explaining the differential expression patterns of genic and intergenic piRNAs. The rationale for sequencing PIWIL2 IPs from adult testes is to capture additional genic piC loci. (C) Read length distributions of the 10dpp testes and PIWIL2 IP small RNA libraries from marmoset, mouse, rat and rabbit, and compared to the adult testes total small RNAs in other primates and mouse and rabbit. (D) Genome browser snapshots of piRNA clusters conserved in Glires and which exhibit stronger piRNA expression in 10dpp testes, piC-Abl2 (left); and piC-Elk4 (right). Purple arrows point to the start of bulk of piRNAs. Plus strand read peaks are red, minus strand read peaks are blue. (E-I)Venn diagrams the number of genic piC loci from mouse, rat and rabbit testes in each of the different stages and samples (E), and comparing between the species for a given stage or sample (F). Overlapping areas are genic piC loci shared between the two libraries. (G) The overlap of Glire species genic piC loci in all samples, between human and mouse, and between Primates and Glires. (H) Overlap of genic piC loci and gene expression profiles from adult testes from human, mouse and dog, using piRNA and expression cutoffs that are twice as stringent as in Fig 2G, right-most set of diagrams. (I) Overlap of identical genic piC loci called by our pipeline from two independently-constructed small RNA libraries from human and chicken adult testes. (J) Glires-focused heatmap of the top 100 genic piC loci ranked by piRNA expression in mouse. (K) Primates-focused heatmap of the top 100 genic piC loci ranked by piRNA expression in human. (L) Estimation of the gain and loss of genic piC loci along each branch of the phylogeny between eleven mammals and the chicken, with the effective gain rates shown in red. (TIF) [file pgen.1005652.s005.tif]

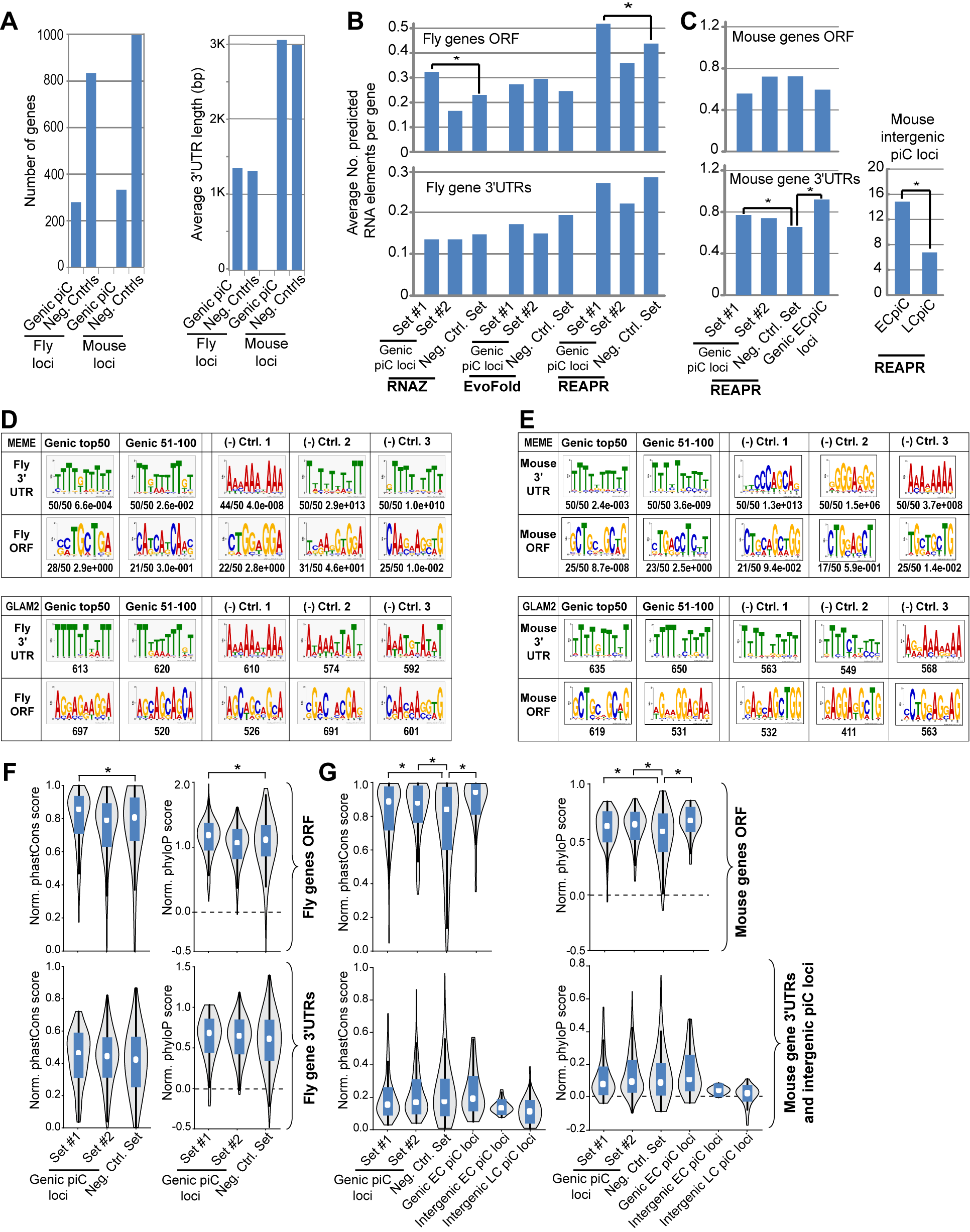

Supplement: S5 Fig — (A) Graph displaying similar numbers of genic piC loci and similar average 3’UTR lengths in between the genic piC loci and the negative control genes for the fly and mouse sets, which do not generate piRNAs. Comparison of the average number of predicted conserved structural elements residing in genic piC transcripts versus transcripts that do not generate piRNAs for fly genes (B) and mouse genes (C). Set#1 is the top half of the genic piC loci with the highest piRNA counts, Set#2 is the bottom half of the genic piC loci with lower piRNA counts. Asterisks mark p<0.01, T-test. Summary of top scoring sequence motifs on fly genes (D) and mouse genes (E) from MEME and GLAM2 analyses. The MEME output scores report the number of transcripts out of the total query containing the top motif and the associated p-value, while the GLAM2 scores reflect the reproducible discovery of the motif during iteration steps in the algorithm. Violin plots of normalized phastCons and phyloP scores for each gene segment (ORF versus 3’UTR) being compared between genic piC loci and negative control genes for fly (F) and mouse (G). Set#1 is the top half of the genic piC loci with the highest piRNA counts, Set#2 is the bottom half of the genic piC loci with lower piRNA counts. Asterisks mark p<0.05, Mann-Whitney test. ECpiC loci Eutherian-Conserved piRNA clusters, LCpiC loci, Less-Conserved piRNA clusters. (TIF) [file pgen.1005652.s006.tif]

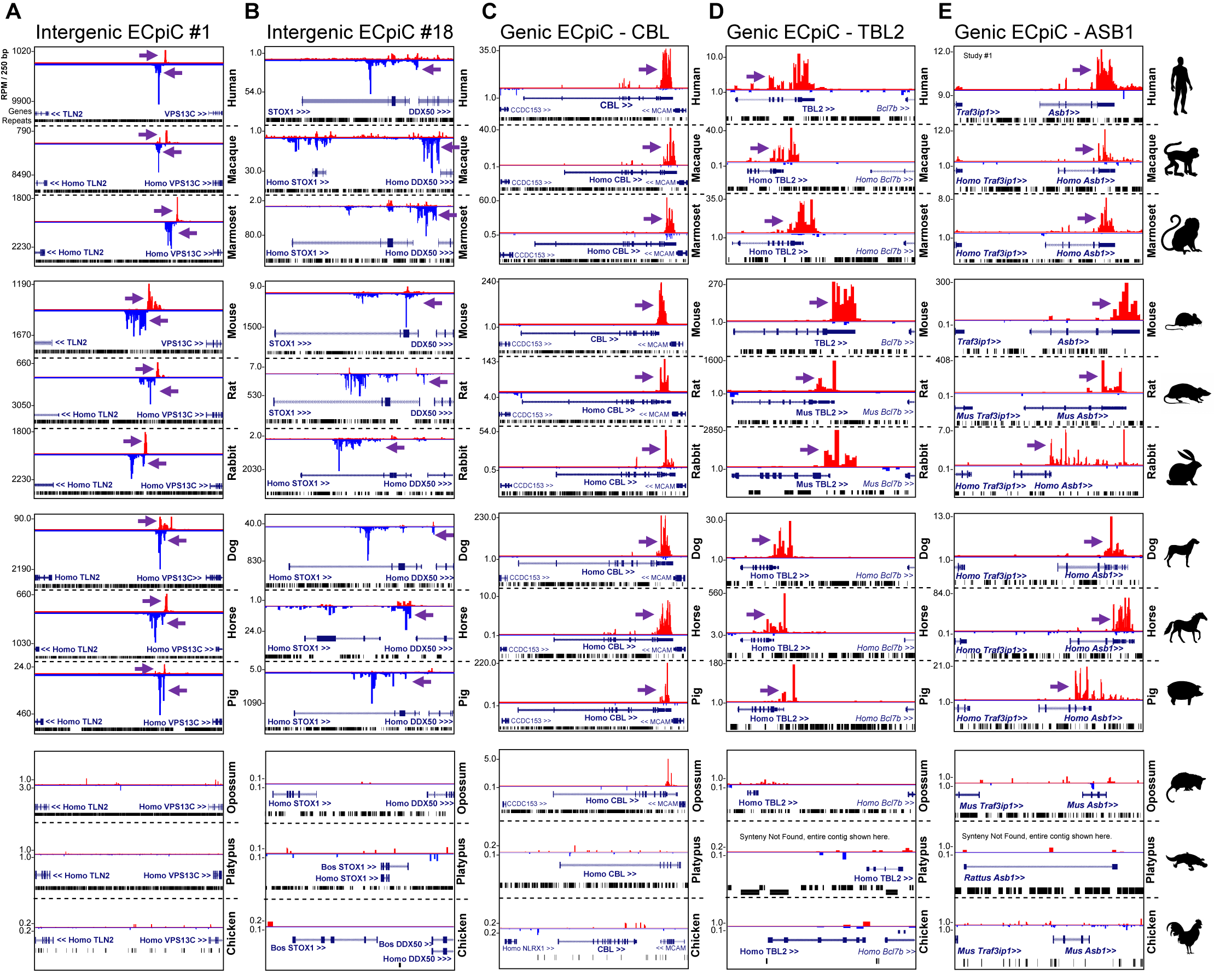

Supplement: S6 Fig — The full compilation across 11 mammals and the chicken of two intergenic ECpiC loci shown in (A) and (B), and three genic ECpiC loci shown in (C), (D) and (E). The majority of piRNAs from intergenic ECpiC #18 are actually antisense to the protein-coding transcript of the STOX1 gene. Plus strand read peaks are red, minus strand read peaks are blue. Purple arrows point to the start of bulk of piRNAs. (TIF) [file pgen.1005652.s007.tif]
