# Supplementary material for: Conserved piRNA Expression from a Distinct Set of piRNA Cluster Loci in Eutherian Mammals
Source: PLoS Genet. 2015 Nov 20;11(11):e1005652. doi: 10.1371/journal.pgen.1005652 (PMC4654475; doi:10.1371/journal.pgen.1005652)
Supplement: S1 Table — Shaded lines are small RNA libraries. Accessions and library citations are listed on the right most columns and at the bottom of the table. (PDF) [file pgen.1005652.s008.pdf]

TABLE S1. LIBRARY SEQUENCING AND ANALYSIS STATISTICS

	DROSOPHILIDS							18,217,233			New libraries made for this study: GSE62556		
	**Library**	**Total Reads**	**read_clipped**	**read_noclipped**	**virus**	**mirna**	**structural RNA**	**genome_mapped**	**not_mapping**	**norm_base**	**GEO/SRA Source for fastq file**	**Reference**	**Genome build**
1	Dmel_FC_sRNA	12,306,325	9,953,274	78,058	62	217,278	6,302,069	1,536,736	2,056,451	1,536,736	GSM1528798	this study	Dm3
2	Dmel_OSScell_s2768_sRNA	24,742,206	20,558,955	649,121	666,270	3,275,114	2,602,831	11,673,018	4,411,155	11,673,018	GSM385744, GSM385748, GSM385821, GSM385822	this study	Dm3
3	Dere_FC_sRNA	11,303,950	9,758,809	55,959	0	609,174	3,222,312	3,109,318	3,090,847	3,109,318	GSM1528801	this study	De1
4	Dyak_FC_sRNA	17,017,729	12,309,436	109,291	12	47,123	5,620,805	5,746,492	1,030,023	5,746,492	GSM1528802	this study	Dy2
5	Dvir_FC_sRNA	15,666,598	12,594,708	61,329	50	76,313	8,168,268	1,842,612	2,598,823	1,842,612	GSM1528803	this study	Dv2
6	Dmel_FC_mRNA	33,510,652			9,933	384	9,182,984	12,960,025	11,357,326	12,960,025	GSM1323913	this study	Dm3
7	Dere_FC_mRNA	27,550,790			1,008	67	8,964,141	10,348,433	8,237,159	10,348,433	GSM1528804	this study	De1
8	Dyak_FC_mRNA	24,308,648			1,456	50	3,248,037	15,604,133	5,454,983	15,604,133	GSM1323914	this study	Dy2
9	Dvir_FC_mRNA	22,075,885			9,579	0	2,730,610	5,744,097	13,591,599	5,744,097	GSM1528805	this study	Dv2
	**GLIRES**												
	**Library**	**Total Reads**	**read_clipped**	**read_noclipped**	**virus**	**mirna**	**structural RNA**	**genome_mapped**	**not_mapping**	**norm_base**	**GEO/SRA Source for fastq file**		
10	Mouse_Adult_testes_sRNA	13,039,474	9,667,162	263,819		1,924,716	780,509	6,200,766	1,026,795	6,200,766	GSM475281	Robine et al, 2009.	Mm10
11	Mouse_10dpp_testes_sRNA	11,277,390	11,007,753	188,238		1,407,590	908,909	4,540,225	4,360,461	4,540,225	GSM1528806	this study	Mm10
12	Mouse_Adult_testes_Mili_IP_sRNA	12,866,614	10,622,491	277,379		317,778	318,017	9,536,909	730,819	9,536,909	GSM475280	Robine et al, 2009.	Mm10
13	Mouse_Adult_testes_Miwi_IP_sRNA	11,743,853	9,918,668	428,317		798,981	401,863	8,125,297	1,024,952	8,125,297	GSM475279	Robine et al, 2009.	Mm10
14	Mouse_Adult_testes_Asb1_ao31(Het)_sRNA	38,062,356	27,410,382	269,697		4,452,772	2,730,396	18,290,894	2,223,963	18,290,894	GSM1528807	this study	Mm10
15	Mouse_Adult_testes_Asb1_ao32(KO)_sRNA	37,593,093	30,322,626	533,765		3,382,935	1,299,818	23,049,908	3,133,493	23,049,908	GSM1528808	this study	Mm10
16	Mouse_Adult_testesAsb1_ao34(Het)_sRNA	48,282,481	39,737,119	318,997		3,862,895	2,747,188	30,246,347	3,222,368	30,246,347	GSM1528809	this study	Mm10
17	Mouse_Adult_testes_Asb1_ao36(KO)_sRNA	54,947,527	39,073,191	258,872		4,042,589	4,948,877	27,446,910	2,938,623	27,446,910	GSM1528810	this study	Mm10
18	Mouse_Adult_Testes_Asb1_AO31(Het)_mRNA	7,072,474						2,391,911		2,391,911	GSM1528811	this study	Mm10
19	Mouse_Adult_Testes_Asb1_AO33(KO)_mRNA	13,548,977						7,581,166		7,581,166	GSM1528812	this study	Mm10
20	Mouse_Adult_Testes_Asb1_AO34(Het)_mRNA	6,315,289						3,211,884		3,211,884	GSM1528813	this study	Mm10
21	Mouse_Adult_Kidney_Asb1_AO34(Het)_mRNA	10,867,990						8,320,434		8,320,434	GSM1528814	this study	Mm10
22	Mouse_Adult_Testes_Asb1_AO36(KO)_mRNA	5,184,354						3,919,633		3,919,633	GSM1528815	this study	Mm10
23	Mouse_Adult_Kidney_Asb1_AO36(KO)_mRNA	7,871,420						3,044,779		3,044,779	GSM1528816	this study	Mm10
24	Mouse_10dpp_testes_mRNA_A	65,998,685						19,667,485		19,667,485	GSM1083628	Margolin et al. 2014	Mm10
25	Mouse_10dpp_testes_mRNA_B	46,904,481						16,725,299		16,725,299	GSM1083629	Margolin et al. 2014	Mm10
26	Mouse_Adult_testes_mRNA_(A)	50,122,297						21,414,799		21,414,799	GSM1083633	Margolin et al. 2014	Mm10
27	Mouse_Adult_testes_mRNA_(B)	67,552,255						28,929,639		28,929,639	GSM1083634	Margolin et al. 2014	Mm10
28	Rat_Adult_testes_sRNA	15,339,599	9,780,902	1,891,488		243,414	142,630	9,021,782	2,264,833	9,021,782	GSM1528817	this study	Rn6
29	Rat_Adult_testes_Mili_IP_sRNA	4,168,157	3,079,568	216,443		92,396	1,088,619	1,161,104	975,226	1,161,104	GSM1528818	this study	Rn6
30	Rat_10dpp_testes_sRNA (merged)											this study	Rn6
31	Rat_10dpp_testes_sRNA (merged)	14,680,154	13,731,790	337,174		2,411,733	216,068	2,596,617	8,861,335	2,596,617	GSM1528819	this study	Rn6
32	Rat_14dpp_testes_mRNA_A	38,786,378						35,113,319		35,113,319	GSM1328757	Yu et al. 2014	Rn6
33	Rat_14dpp_testes_mRNA_B	46,510,409						42,083,939		42,083,939	GSM1328758	Yu et al. 2014	Rn6
34	Rat_14dpp_testes_mRNA_C	55,939,035						50,592,474		50,592,474	GSM1328759	Yu et al. 2014	Rn6
35	Rat_14dpp_testes_mRNA_D	40,535,357						36,844,407		36,844,407	GSM1328760	Yu et al. 2014	Rn6
36	Rat_Adult_testes_mRNA_A	44,266,539						38,505,291		38,505,291	GSM1328765	Yu et al. 2014	Rn6
37	Rat_Adult_testes_mRNA_B	43,885,804						38,176,147		38,176,147	GSM1328766	Yu et al. 2014	Rn6
38	Rat_Adult_testes_mRNA_C	53,823,609						46,724,087		46,724,087	GSM1328767	Yu et al. 2014	Rn6
39	Rat_Adult_testes_mRNA_D	48,442,971						41,953,284		41,953,284	GSM1328768	Yu et al. 2014	Rn6
40	Rabbit_Adult_testes_Mili_IP_sRNA	6,939,588	4,501,993	39,583		349,121	1,334,741	2,254,486	603,392	2,254,486	GSM1528820	this study	Oc2
41	Rabbit_Adult_testes_sRNA (merged)											this study	Oc2
42	Rabbit_Adult_testes_sRNA (merged)	9,393,800	8,987,653	159,487		113,614	128,055	7,411,815	1,493,702	7,411,815	GSM1528821	this study	Oc2
43	Rabbit_Testes_mRNA_Lau	8,688,620						3,959,109		3,959,109	GSM1528822	this study	Oc2
44	Rabbit_Testes mRNA SRX110711_A	43,130,924						22,021,929		22,021,929	SRR388292	public but not published	Oc2
45	Rabbit_Testes mRNA SRX110711_B	43,480,016						20,004,916		20,004,916	SRR388295	public but not published	Oc2
46	Rabbit_Testes mRNA SRX110711_C	42,338,840						21,831,038		21,831,038	SRR388300	public but not published	Oc2
	**PRIMATES**												
	**Library**	**Total Reads**	**read_clipped**	**read_noclipped**	**virus**	**mirna**	**structural RNA**	**genome_mapped**	**not_mapping**	**norm_base**	**GEO/SRA Source for fastq file**		
47	Human_Adult_Testes_sRNA_1	55,049,226	38,224,512	1,301,272		356,665	11,529,738	24,527,637	449,472	24,527,637	SRP021475 (SRR835325, SRR835324, SRR950451)	Ha et al, 2014	Hg38
48	Human_Adult_Testes_sRNA_2	15,118,197	14,318,593	49,914		4,419,743	479,097	9,044,130	49,914	9,044,130	ERP003784 (ERR328151)	Yang et al, 2013	Hg38
49	Human_Adult_Testes_mRNA	63,348,292						35,774,839	27,573,453	35,774,839	GSM759886		Hg38
50	Macaque_Adult_Testes_sRNA	15,051,771	10,213,025	4,431,787		670,359	63,423	7,340,939	4,408,618	7,340,939	GSM678415	Yan et al, 2011	Rm3
51	Macaque_Adult_Testes_mRNA_A	10,453,892						10,412,358	41,534	10,412,358	GSM1229147	public but not published	Rm3
52	Macaque_Adult_Testes_mRNA_B	9,681,604						9,646,521	35,083	9,646,521	GSM1229148	public but not published	Rm3
53	Marmoset_Adult_Testes_sRNA	18,832,666	17,739,012	321,676		1,045,789	1,221,019	14,106,109	321,671	14,106,109	GSM1278256	Hirano et al, 2014	Cj3
54	Marmoset_Piwil1_IP_sRNA	140,662,017	139,815,057	283,392		354,857	7,953,980	116,979,055	283,392	116,979,055	GSM1278257	Hirano et al, 2014	Cj3
55	Marmoset_Adult_Testes_mRNA	168,398,076						31,609,181	136,788,895	31,609,181	GSM1278258	Hirano et al, 2014	Cj3
	**LAURASIATHERIANS**												
	**Library**	**Total Reads**	**read_clipped**	**read_noclipped**	**virus**	**mirna**	**structural RNA**	**genome_mapped**	**not_mapping**	**norm_base**	**GEO/SRA Source for fastq file**		
56	Dog_Adult_Testes_sRNA	65,893,540	65,224,405	361,824				61,241,147	157,607	61,241,147	GSM1152898 (SRR871530)	Platt et al. 2014	Cf3
57	Dog_Adult _Tests_mRNA	68,310,732						31,818,021	36,492,711	31,818,021	SRX111069 (SRR388746, SRR388755, SRR388758)	Hoeppner et al, 2014	Cf3
58	Horse_Adult_Testes_sRNA	69,420,477	68,305,099	507,608				61,357,882	176,456	61,357,882	GSM1152900(SRR871532)	Platt et al. 2014	Ec2
59	Horse_Adult_Testes_mRNA_A	11,435,000						2,026,169	9,408,831	2,026,169	GSM1139276(SRR850301)	Coleman et al. 2013	Ec2
60	Horse_Adult_Testes_mRNA_B	8,404,518						2,222,605	6,181,913	2,222,605	GSM1139276(SRR850302)	Coleman et al. 2013	Ec2
61	Horse_Adult_Testes_mRNA_C	17,310,580						16,695,922	614,658	16,695,922	GSM545509(SRR836407)	Coleman et al. 2013	Ec2
62	Pig_120d_testes_sRNA	14,660,953	13,751,928	453,390				12,449,384	434,539	12,449,384	GSM697624 (SRR139076)	Lian et al. 2012	Ss3
63	Pig_testes_mRNA_A	60,798,185						27,433,113	33,365,072	27,433,113	SRX229760,SRX229761,SRX229762	Gunawan et al, 2013	Ss3
64	Pig_testes_mRNA_B	81,073,003						38,635,926	42,437,077	38,635,926	SRX229765, SRX229766, SRX229767	Gunawan et al, 2013	Ss3
	**OTHER TETRAPODS LIBRARIES**												
	**Library**	**Total Reads**	**read_clipped**	**read_noclipped**	**virus**	**mirna**	**structural RNA**	**genome_mapped**	**not_mapping**	**norm_base**	**GEO/SRA Source for fastq file**		
65	Opossum_Adult_Testes_sRNA_main_mdo_tes	30,914,709	22,720,151	1,651,718				20,023,437	1,618,650	20,023,437	GSM995319 (SRR553591)	Meunier et al, 2013	Md2
66	Opossum testes mRNA_A:	58,535,821						34,011,634	24,524,187	34,011,634	GSM1064849 (SRR649379, SRR649380)	Necsulea et al, 2014	Md2
67	Opossum testes mRNA_B:	49,481,463						23,274,882	26,206,581	23,274,882	GSM752611 + GSM752613 (SRR306755, SRR306756)	Brawand et al, 2011	Md2
68	Platypus_Adult_Testes_sRNA_main_oan_tes	27,624,562	22,376,147	835,079				19,283,798	814,021	19,283,798	GSM995324 (SRR553596)	Meunier et al, 2013	Oa1
69	Platypus testes mRNA_A:	63,274,029						30,518,320	32,755,709	30,518,320	GSM1064852 (SRR649383, SRR649384)	Necsulea et al, 2014	Oa1
70	Platypus testes mRNA_B:	68,800,467						20,728,760	48,071,707	20,728,760	GSM752583 + GSM752585 (SRR306739, SRR306740, SRR306741)	Brawand et al, 2011	Oa1
71	Rooster_Adult_Testis_sRNA_Wild Type_Oxidized	143,448,225	130,820,103	5,093,010				110,547,085	5,093,010	110,547,085	GSM1096613 (SRR772069)	Li et al, 2013	Gg4
72	Rooster_Adult_Tests_sRNA_main_gga_tes	34,200,789	26,393,594	1,117,009				22,384,178	1,085,563	22,384,178	GSM995329 (SRR553601)	Meunier et al, 2013	Gg4
73	Chicken testes mRNA_A:	73,730,209						42,851,146	30,879,063	42,851,146	GSM1064855 (SRR649389, SRR649390)	Necsulea et al, 2014	Gg4
74	Chicken testes mRNA_B:	67,150,271						28,104,303	39,045,968	28,104,303	GSM752567 + GSM752568 (SRR306721, SRR306722, SRR306723)	Brawand et al, 2011	Gg4

Libraries Citations

Robine, N., Lau, N.C., Balla, S., Jin, Z., Okamura, K., Kuramochi-Miyagawa, S., Blower, M.D., and Lai, E.C. (2009). A broadly conserved pathway generates 3' UTR-directed primary piRNAs. Current Biology *19*, 2066-2076.

Margolin, G., Khil, P.P., Kim, J., Bellani, M.A., and Camerini-Otero, R.D. (2014). Integrated transcriptome analysis of mouse spermatogenesis. BMC genomics *15*, 39.

Yu, Y., Fuscoe, J.C., Zhao, C., Guo, C., Jia, M., Qing, T., Bannon, D.I., Lancashire, L., Bao, W., Du, T., *et al.* (2014). A rat RNA-Seq transcriptomic BodyMap across 11 organs and 4 developmental stages. Nature communications *5*, 3230.

Ha, H., Song, J., Wang, S., Kapusta, A., Feschotte, C., Chen, K.C., and Xing, J. (2014). A comprehensive analysis of piRNAs from adult human testis and their relationship with genes and mobile elements. BMC genomics 15, 545.

Yang Q, Hua J, Wang L, Xu B, Zhang H, Ye N, Zhang Z, Yu D, Cooke HJ, Zhang Y, Shi Q. MicroRNA and piRNA profiles in normal human testis detected by next generation sequencing. PLoS One. 2013 Jun 24;8(6):e66809.

Hirano, T., Iwasaki, Y.W., Lin, Z.Y., Imamura, M., Seki, N.M., Sasaki, E., Saito, K., Okano, H., Siomi, M.C., and Siomi, H. (2014). Small RNA profiling and characterization of piRNA clusters in the adult testes of the common marmoset, a model primate. Rna *20*, 1223-1237.

Platt, R.N., 2nd, Vandewege, M.W., Kern, C., Schmidt, C.J., Hoffmann, F.G., and Ray, D.A. (2014). Large numbers of novel miRNAs originate from DNA transposons and are coincident with a large species radiation in bats. Molecular biology and evolution *31*, 1536-1545.

Hoeppner, M.P., Lundquist, A., Pirun, M., Meadows, J.R., Zamani, N., Johnson, J., Sundstrom, G., Cook, A., FitzGerald, M.G., Swofford, R., *et al.* (2014). An improved canine genome and a comprehensive catalogue of coding genes and non-coding transcripts. PloS one *9*, e91172.

Coleman, S.J., Zeng, Z., Hestand, M.S., Liu, J., and Macleod, J.N. (2013). Analysis of unannotated equine transcripts identified by mRNA sequencing. PloS one *8*, e70125.

Lian, C., Sun, B., Niu, S., Yang, R., Liu, B., Lu, C., Meng, J., Qiu, Z., Zhang, L., and Zhao, Z. (2012). A comparative profile of the microRNA transcriptome in immature and mature porcine testes using Solexa deep sequencing. The FEBS journal *279*, 964-975.

Gunawan, A., Sahadevan, S., Neuhoff, C., Grosse-Brinkhaus, C., Gad, A., Frieden, L., Tesfaye, D., Tholen, E., Looft, C., Uddin, M.J., *et al.* (2013). RNA deep sequencing reveals novel candidate genes and polymorphisms in boar testis and liver tissues with divergent androstenone levels. PloS one *8*, e63259.

Meunier, J., Lemoine, F., Soumillon, M., Liechti, A., Weier, M., Guschanski, K., Hu, H., Khaitovich, P., and Kaessmann, H. (2013). Birth and expression evolution of mammalian microRNA genes. Genome research *23*, 34-45.

Necsulea, A., Soumillon, M., Warnefors, M., Liechti, A., Daish, T., Zeller, U., Baker, J.C., Grutzner, F., and Kaessmann, H. (2014). The evolution of lncRNA repertoires and expression patterns in tetrapods. Nature *505*, 635-640.

Brawand, D., Soumillon, M., Necsulea, A., Julien, P., Csardi, G., Harrigan, P., Weier, M., Liechti, A., Aximu-Petri, A., Kircher, M., *et al.* (2011). The evolution of gene expression levels in mammalian organs. Nature *478*, 343-348.

Li, X.Z., Roy, C.K., Dong, X., Bolcun-Filas, E., Wang, J., Han, B.W., Xu, J., Moore, M.J., Schimenti, J.C., Weng, Z., *et al.* (2013). An ancient transcription factor initiates the burst of piRNA production during early meiosis in mouse testes. Molecular cell *50*, 67-81.
