# Supplementary material for: Conserved piRNA Expression from a Distinct Set of piRNA Cluster Loci in Eutherian Mammals
Source: PLoS Genet. 2015 Nov 20;11(11):e1005652. doi: 10.1371/journal.pgen.1005652 (PMC4654475; doi:10.1371/journal.pgen.1005652)
Supplement: S5 Table — (PDF) [file pgen.1005652.s012.pdf]

Table S5. Oligonucleotides used in this study.

Oligonucloetides for Northern blot probes

Oligonucleotide name(s)	Oligonucleotide sequence
Dvir *Adar* piRNAs probes	GCGTCGGAGCAGGGACACAATGTCGTA
	CACCCCAACGATCATGACCAAGGTG
	GCGCATTCCAAAGAGCCACCGTGGGA
Dmel *Tj* piRNAs DNA probes	TCCAAGTTCTCAAACTGATATTCATCGGA
	GCTTCTGTTTCGCTTAATACTAGTAGGGA
	ATTTATTGTCGCAATCAGAATAATGGCA
	TCTGTCGATCACTTTCCAAGTTC
Dmel *Tj* piRNAs RNA/DNA hybrid probes	rArGrUrCAAACTGATAATCrArGrCrA
	rCrUrCrUAGAACAACTATCrGrCrArU
Dmel/Dere *dimunitive* piRNAs hybrid probes	rUrCrCrUCCAAGGATGCGTrCrUrCrU
	rArCrCrAAATCAAATCGCGrCrGrGrA
Dvi *dimunitive* piRNAs RNA/DNA hybrid probes	rCrUrGrCACTCCCATTTCGrCrGrCrA
	rCrUrGrAAGTATGTGTTTGrGrGrCrA
Dmel *Roo* piRNAs probes	TGGGCTCCGTTCATATCTTATG
	TGAGAGTTCGCTATTCGAAGAA
	TCTGAGGCATCCGTTTGGTAAA
Dmel miR-12 miRNA probe	accagtacctgatgtaatactca
Mouse Kctd7piRNAs probes	ACGGCTTTGGCAGCAGCTATGGTTAATCT
	GATCTTCAGAGAGAACCTTTAGAACT
	CTTGACATCTGAGAGGGATCCTACAT
	ACTCAGCCTCTCTGTTACACTCAGAG
Mouse Arhgap20 piRNAs	GTTTTCTTTGTGTCTGGAAAGTGCAAT
	CCTTCTATCAGTATCTGAAAATCTCAGGAA
	CCATGGCACTTTACCCACTAGTTTTCTC
	TTGTCTTTGTATCTCCATTTCAGAAAGATA
Rat Piwil1 piRNAs	GAGAACCTGCCGACTCGGCTGCTTTA
	GAACCTGCGGACTCGGCTGCTTTAGG
	ATCCCAGAGTGGTCTTCTGCAGTTTA
Rabbit Riok1 piRNAs	AAATTCAGTCAGATTCTCCAAATTCA
	GCATTCCACATTTCATCTCGTTTCTGAGC
	GCTGTATAAACCATAACCCATTAACAGGA
	GCTAACAACCATTTCTGACAAAGTATA
Mouse Cbl piRNAs probes	CGATTCGCAAAAGCTGTCTAGTTTGTAAATTA
	CGTCTGAGAAGGCCAGTAGCCTTCTGATTCTCA
	CGCCTATTTCTACTTCTACAGAGCCGTTAAGA
	ACAAGAACCAAGTCTTCCATGCATTCCTA
Mouse Asb1 piRNAs probes	TTGAAAAATGCCAATTATCTACTTTACCA
	AACATGCAGAACCTTGAAGTGAATTGAAAGG
	TCAGTCCATGCCGATGCTGTGTCACATTCA
	GATCACTGCCCACTTCAAAGCTTGCA

Oligonucleotides for RT-qPCR

Oligonucleotide name(s)	Oligonucleotide sequence
Mm Asb1 Forward primer	ATGGCGGAGGGCGGGACC
Mm Asb1 Reverse primer	AGCCACTCCTTCAGATTAGGAC
Neo Forward primer	AGACAATCGGCTGCTCTGAT
Neo Reverse primer	TGCATCAGCCATGATGGATAC

Oligonucleotides for libraries construction

Oligonucleotide name(s)	Oligonucleotide sequence
Biotinylated DNA oligo antisense to 2S rRNA	TACAACCCTCAACCATATGTAGTCCAAGCATACAACC CTCAACCATATGTA GTCCAAGCAGTCGA-3′biotin
3' adaptor linker	p<u>CG</u>TCGTATGCCGTCTTCTGCTTGT/3AmMO/
5' adaptor linker	GTTCAGAGTTCTACAGTCCGACGATCNNNrXrXrXrArA
18.206 (RT and PCR primer)	CAAGCAGAAGACGGCATA
44.45 (forward PCR primer)	AATGATACGGCGACCACCGACAGGTTCAGAGTTCTA CAGTCCGA
